# Evaluation of mesenchymal stem cells as an *in vitro* model for inherited retinal diseases

**DOI:** 10.3389/fcell.2024.1455140

**Published:** 2024-11-15

**Authors:** Maria Dodina, Dzerassa Gurtsieva, Alexander Karabelsky, Ekaterina Minskaia

**Affiliations:** Sirius University of Science and Technology, Krasnodar, Russia

**Keywords:** mesenchymal stem cells, MSC, inherited retinal diseases, IRD, *in vitro* disease modeling, retinal cells

## Abstract

Retinal pathologies are major causes of vision impairment and blindness in humans, and inherited retinal diseases (IRDs), such as retinitis pigmentosa, Leber congenital amaurosis, and Stargardt disease, greatly contribute to this problem. *In vitro* disease modeling can be used for understanding the development of pathology and for screening therapeutic pharmaceutical compounds. In the preclinical research phase, *in vitro* models complement *in vivo* models by reducing animal studies, decreasing costs, and shortening research timelines. Additionally, animal models may not always accurately replicate the human disease phenotype. This review examines the types of cells that can be used to create *in vitro* IRD models, including retina-specific cell lines, primary retinal cells, induced pluripotent stem cells (iPSCs), and more. Special attention is given to mesenchymal stem cells (MSCs), which are characterized by various isolation sources, relative ease of isolation, and straightforward differentiation. MSCs derived from bone marrow (BM), adipose tissue (AT), dental tissue (DT), umbilical cord (UC), and other sources can differentiate into retinal cells, including photoreceptor cells and retinal pigment epithelial (RPE) cells, dysfunction of which is most commonly associated with IRDs. Subsequent differentiation of MSCs into retinal cells can be carried out via various methods: culturing in induction media supplemented with certain growth factors, co-culturing with retinal cells or in their conditioned media, or regulating gene expression with viral vector-delivered transcription factors (TFs) or microRNAs (miRNAs). Compared to the popular iPSCs, for example, MSC-based models are significantly cheaper and faster to obtain, making them more feasible for large-scale drug screening. Nevertheless, the existing differentiation methods need further optimization for this promising platform to receive the success it deserves.

## 1 Introduction

The mammalian retina is a structurally complex compartment of the eye, consisting of over 60 types of cells with diverse functions (Masland, 2012). The choroid, the retinal pigment epithelium (RPE), and the Bruch’s membrane comprise the outer blood-retinal barrier (BRB), which selectively regulates the entry of various substances from the choroidal blood capillaries to the retinal cells (Nickla and Wallman, 2010; Fields et al., 2020). RPE cells play a crucial role in supporting the function of photoreceptors by participating in the transport of nutrients and metabolic waste products from these cells, phagocytizing their outer segments, and regenerating visual pigment (Fuhrmann et al., 2014). Their cytoplasm contains numerous pigment granules, containing melanin, which plays a photoprotective role (Istrate et al., 2020). Importantly, RPE cells secrete various growth factors and other proteins essential for the function of photoreceptors and choroidal blood capillary cells (Kay et al., 2013), which include immunosuppressive molecules that provide immune privilege to the eye (Ishida et al., 2003).

**FIGURE 1:**
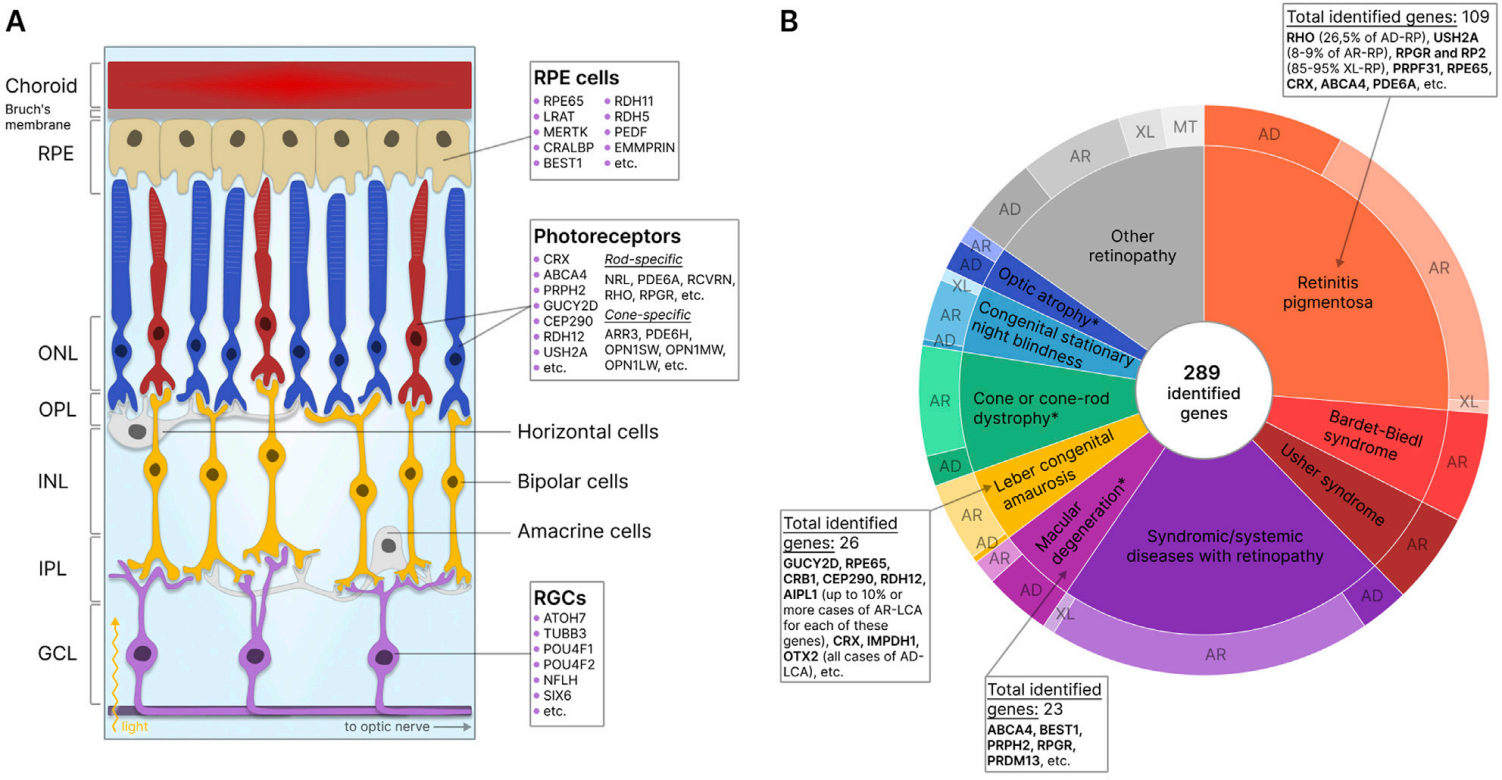
Retinal structure and IRD-associated genes expressed in retinal cells. **(A)** Structure of the retina showing major cells and layers. RPE - retinal pigment epithelium; RGCs - retinal ganglion cells; ONL - outer nuclear layer; OPL - outer plexiform layer; INL - inner nuclear layer; IPL - inner plexiform layer; GCL - ganglion cell layer. Genes specific to photoreceptors, RPE, and RGCs are indicated. **(B)** IRD-associated genes by disease category. The data for the diagram was obtained from RetNet (Retinal Information Network, 2024). While different mutations in the same gene may be associated with different diseases, each identified gene is counted only once for the first-reported disease (usually the most common disease). The total number of identified genes and the most commonly disease-associated genes are listed by the disease category. The outer circle of the diagram reflects the number of genes associated with different types of inheritance: AD - autosomal dominant; AR - autosomal recessive; XL - X-linked; MT - mitochondrial. *- diseases for which XL-forms are also identified.

In the retina itself, three layers of cells can be distinguished: the outer nuclear layer (ONL), the inner nuclear layer (INL), and the ganglion cell layer of the retina (GCL). These cell layers are separated by the outer (OPL) and the inner (IPL) synaptic (plexiform) layers (Kolb et al., 2001). The ONL comprises photoreceptor cells, light-sensitive cells responsible for the conversion of light signals into electrochemical impulses. The two types of photoreceptors include rods and cones. Rod photoreceptors contain the light-sensitive pigment rhodopsin and are responsible for vision under dim illumination, whereas cone photoreceptors contain one of three types of opsins (red, green, and blue) as pigments and provide high-resolution daytime color vision (Salesse, 2017; Schmidt et al., 2019; Lamb, 2022). The electrochemical signal is transmitted from photoreceptors to bipolar cells, other retinal nerve cells located in the INL (Euler et al., 2014), and then to retinal ganglion cells (RGC) of GCL, which then direct the signal to the brain through axons forming the optic nerve (Sanes and Masland, 2015). The transmission of signals from photoreceptors to bipolar cells is modulated by horizontal cells in the OPL (Poché and Reese, 2009) and from bipolar cells to ganglion cells by amacrine cells in the IPL (Zhang C. and McCall, 2012). In addition, mammalian retinas also contain glial cells of three main types: Müller cells, astrocytes, and microglia, which provide structural and functional support to retinal neurons (Vecino et al., 2016). A schematic presentation of the retina showing the location of the described cell types is shown in Figure 1A.

The degeneration of the major types of retinal cells results in the loss of their functions and disruption of the structural integrity of the retina, leading to serious visual impairment or, in the absence of treatment, complete blindness. Retinal diseases can be classified into inflammatory, degenerative, vascular, and hereditary conditions. IRDs, genetically and phenotypically heterogeneous conditions associated with mutations in one or more genes, most commonly lead to the degeneration of photoreceptor and RPE cells. Currently, about 290 genes are known to contain mutations that result in the disruption of development, loss of function, or death of these cells (Retinal Information Network, 2024) (Figure 1B). The expression of mutant proteins negatively impacts visual cycle pathways, phototransduction, and the maintenance of retinal cell viability (Manley et al., 2023). Studies indicate that 36% of the global population (2.7 billion individuals) are healthy carriers of at least one mutation associated with autosomal recessive IRDs (AR-IRDs) (Hanany et al., 2020). Mutations leading to autosomal dominant IRDs (AD-IRDs) are typically less common, tend to be less severe in phenotype, and manifest at a later age (Hanany and Sharon, 2019). In addition, rare X-linked (XL-IRDs) and mitochondrial (MT-IRDs) IRDs have also been identified (De Silva et al., 2021; Zeviani and Carelli, 2021).

Diseases associated with the degeneration of photoreceptors are among the most common within IRDs and are usually characterized by the primary loss of one type of photoreceptor cell - rods or cones - and the secondary loss of the other (rod-cone and cone-rod dystrophies). Retinitis pigmentosa (RP), for example, falls under the category of rod-cone dystrophy, marked by the loss of photoreceptor cells and the appearance of pigment deposits on the retina (Verbakel et al., 2018). Most forms of RP are inherited in autosomal dominant (AD-RP), autosomal recessive (AR-RP), and X-linked (XL-RP) manners, with XL-RP considered the most severe form. Due to the diversity of inheritance patterns, the disease exhibits wide heterogeneity: over 100 genes are associated with RP, yet in half of all cases, the genetic etiology remains unknown. AD-RP is most commonly linked to mutations in the rhodopsin (26.5% of all cases), AR-RP to mutations in the USH2A (8%–9% of all cases), and XL-RP to mutations in the RPGR and RP2 (85%–95% of cases) genes (Bhardwaj et al., 2022). Syndromic forms of RP, such as Usher, Bardet-Biedl, and Senior-Loken syndromes, characterized by the presence of concurrent non-ocular conditions, are also known (Liu et al., 2010).

An illustrative example of cone-rod dystrophy is Leber congenital amaurosis (LCA), a severe form of retinal dystrophy with early onset, primarily inherited in an autosomal recessive manner. While mutations in about 30 genes were identified as the primary cause of LCA, most commonly (with a frequency of up to 10% or more), this condition is linked to mutations in the GUCY2D, RPE65, CRB1, CEP290, and RDH12 genes (Huang C. H. et al., 2021).

Inherited macular degenerations comprise a significant group of IRDs (Kelly and Maumenee, 1999), with Stargardt disease (SD) being the most prevalent. SD, characterized by gradual central vision loss and RPE cell degeneration associated with lipofuscin accumulation, is most often inherited in an autosomal recessive manner and is caused by mutations in the ABCA4 gene (Huang D. et al., 2022). This group also includes diseases such as Best vitelliform macular dystrophy (BVMD) and North Carolina macular dystrophy (NCMD), typically inherited in an autosomal dominant manner (Tsang and Sharma, 2018a; 2018b). Other examples of IRDs include choroideremia (Sarkar and Moosajee, 2022), X-linked retinoschisis (XLRS) (Ku et al., 2023), Fundus albipunctatus (Yamamoto et al., 1999), and Malattia Leventinese (ML) disease (Vaclavik, 2020), among many others. Often, different mutations within the same gene lead to the development of phenotypically diverse retinal diseases. Interestingly, some mutations in certain genes lead not only to visual impairment, but also to dysfunction in other organs. For example, Batten disease, also known as neuronal ceroid lipofuscinoses, constitutes a family of devastating lysosomal storage disorders that lead to deterioration of vision, cognitive and motor functions, and premature death (Johnson et al., 2019). And finally, multifactorial retinal diseases such as age-related macular degeneration (AMD), influenced by genetic predisposition among other factors, also exist (Heesterbeek et al., 2020).

The identification of IRD-associated mutations facilitated the development of *in vivo* animal models for studying disease pathogenesis and testing new pharmaceutical treatments. The most relevant *in vivo* IRD models are those involving non-human primates (NHPs) because their retinas are anatomically and physiologically similar to those of humans. However, developing these models is a complex and expensive task (Seah et al., 2022). While other *in vivo* IRD models such as cats, dogs, and pigs are known (Moshiri, 2021), rodent models (typically mice) are used more frequently due to their small size, short lifespan, and cost-effectiveness (Dalke and Graw, 2005; Collin et al., 2020). However, mouse models often fail to mimic human retinal diseases due to the substantial difference between mouse and human retinas. The mouse retina is thinner and lacks a cone-rich region with high visual acuity equivalent to the human macula. Additionally, mouse cones express only two types of opsins, sometimes simultaneously (Neitz M. and Neitz J., 2001; Volland et al., 2015). For instance, mutations in retinol dehydrogenase (RDH) genes in humans lead to severe retinal dystrophies: RDH5 is associated with Fundus albipunctatus (Yamamoto et al., 1999), RDH8 with SD (Zampatti et al., 2023), and RDH12 with LCA type 13 or RP (Sarkar and Moosajee, 2019). However, mice with knockouts of these genes typically exhibit a mild disease phenotype without pronounced retinal degeneration, manifesting primarily as delayed adaptation to darkness (Kurth et al., 2007; Maeda et al., 2007). The exact reason why these mice do not develop the pathological phenotype observed in humans is unknown, but it is hypothesized that additional RDHs in rodents may compensate for the lost enzymes. Similarly, most mouse models with Usher syndrome mutations demonstrate hearing loss but not visual impairment (Géléoc and El-Amraoui, 2020; Stemerdink et al., 2022), possibly due to the underdeveloped photoreceptor periciliary membrane and the absence of the calyceal structure in mouse photoreceptor outer segments compared to humans (Sahly et al., 2012).

Due to the above-mentioned limitations of *in vivo* models, the major one of which is the inaccurate presentation of the disease phenotype, there is an obvious need for effective *in vitro* IRD models that can be used to study the healthy physiology of retinal cells, the IRD pathology, and to test various drugs. The *in vitro* models are especially important in the early stages of preclinical research for rapid and routine screening of therapeutic molecules and have a great advantage as they reduce the number of animal experiments, making research more ethically acceptable, cost-effective, and fast.

There are two main types of retinal disease models: two-dimensional (2D) and three-dimensional (3D) (Alfonsetti et al., 2021; Schnichels et al., 2021; Zhu Y. et al., 2022). 2D cultures typically consist of a monolayer of cells, whereas 3D cultures are multilayered. Although 2D cultures cannot replicate the tissue structure and cell interactions inherent to tissues, they are widely used in research due to their ease of maintenance, low cost, high reproducibility, and suitability for long-term and large-scale experiments. Emerging 3D cultures, known as organoids, are multilayered and can reproduce some *in vivo* cell interactions. However, most current retinal organoids (ROs) cannot accurately replicate the organ morphology, and their production is expensive, time-consuming, and characterized by insufficient reproducibility due to significant heterogeneity (Jensen and Teng, 2020). Nonetheless, ROs technology holds great promise for modeling retinal diseases (Cheng and Kuehn, 2023; Liang et al., 2023; Kurzawa-Akanbi et al., 2024).

The most promising approach to creating *in vitro* IRD models relies on stem cells of various origins (Achberger et al., 2019). Special attention is given to pluripotent stem cells (PSCs), iPSCs, and embryonic stem cells (ESCs) due to their ability to form organoid 3D retinal structures. However, more accessible adult stem cells, particularly MSCs, are relevant for creating 2D retinal models due to their numerous sources, relative cost-effectiveness, and ease of differentiation control. MSCs can potentially serve as a source for various *in vitro* models due to their ability to differentiate into multiple cell types: chondrocytes, osteoblasts, adipocytes, hepatocytes, cardiomyocytes, neurons, and more (Afflerbach et al., 2020). For example, MSCs differentiated into hepatocyte-like cells can be used to assess hepatotoxicity (Cipriano et al., 2017); MSCs differentiated into motor neurons, astrocytes, and oligodendrocytes can be useful for modeling neurodegenerative brain diseases (Brodie and Slavin, 2013); and MSCs differentiated into lung epithelial cells can be used to create 3D lung structures via bioprinting (da Rosa et al., 2023). Currently, MSCs are widely used in cell replacement therapy for retinal diseases (Adak et al., 2021), but their potential for creating retinal disease cell models remains underappreciated.

Successful *in vitro* IRD modeling certainly remains an unresolved problem, and it is important to consider various approaches balancing their advantages and disadvantages. Therefore, the present review focuses on the following: i) various cell types used for *in vitro* IRD modeling: immortalized cell lines, primary cells, multipotent stem cells, iPSCs, and MSCs; ii) the various features of MSCs, such as multiple isolation sources and ease of differentiation, among others, that make these cells so useful for IRD modeling; and iii) examples of clinical trials using MSCs for the treatment of retinal diseases.

## 2 Types of cells used for *in vitro* IRD modeling

**FIGURE 2:**
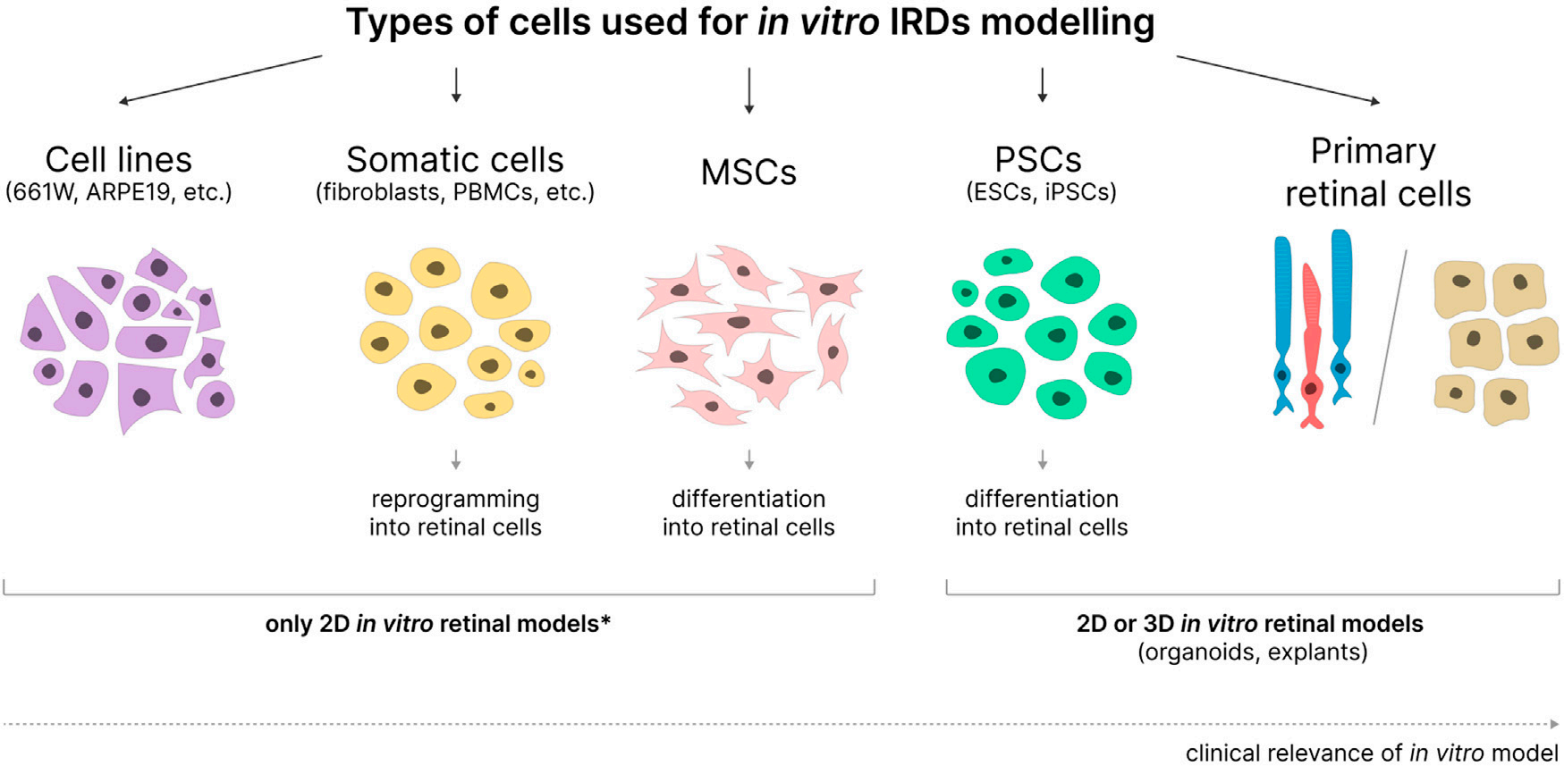
Cell types used in the development of *in vitro* IRD models. MSCs - mesenchymal stem cells; PSCs - pluripotent stem cells; ESCs–embryonic stem cells; iPSCs–induced pluripotent stem cells. 2D or 3D models can be obtained based on various cell types. *- applicable when not used in combination with organ-on-a-chip and bioprinting technologies.

Immortalized cell lines, primary retinal cells, somatic cells, MSCs, and PSCs reprogrammed or differentiated into cells similar to retinal cells can all be used for *in vitro* modeling of IRDs with various degrees of success (Figure 2).

### 2.1 Immortalized cell lines

Cell immortalization can be achieved by preventing replicative senescence, which occurs due to the disruption of cell cycle checkpoints (such as p53, p16, pRb, etc.), the regulation of telomerase expression, or the activation of certain oncogenes. Immortalized cell lines can be generated via various methods: deriving lines from primary tumor cells, transducing non-tumor cells with viral vectors delivering oncogenic viral genes (such as SV40, HPV, or EBV), enforcing the expression of key immortality proteins (such as telomerase), and more (Yeager and Reddel, 1999). Immortalized cell lines can divide indefinitely, are homogeneous, relatively inexpensive, accessible, and easy to sustain. The primary drawback of these cell lines is that they often significantly differ in function and morphology from their original *in vivo* counterparts due to genetic and epigenetic alterations that occur during immortalization, leading to changes in cellular metabolism (Maqsood et al., 2013). Consequently, creating clinically relevant *in vitro* disease models based on these cells can be rather challenging. Nevertheless, they are still widely used for studying healthy physiology and pathological processes, as well as initial drug screening.

Since IRDs are primarily associated with dysfunctions in photoreceptor and RPE cells, photoreceptor-like and RPE-like cell lines are most important for disease modeling. The 661W cell line, derived from a retinal tumor in a transgenic mouse line expressing the SV40 large T-antigen under the control of the interphotoreceptor retinoid-binding protein (IRBP) promoter, closely resembles photoreceptor cells. These cells express markers of cone photoreceptors (blue and green opsins, cone transducin, and cone arrestin) and have a neuronal cell-like morphology but lack structures analogous to photoreceptor inner and outer segments (Tan et al., 2004). Later studies demonstrated that, in addition to cone-specific proteins, such as phosphodiesterase PDE6H, 661W cells express certain rod-specific proteins, such as rod phosphodiesterase PDE6B, but do not express rhodopsin (Mencl et al., 2018). Currently, the 661W cell line is widely used for studying macular degenerations (Kuse et al., 2017; Terao et al., 2019; Song et al., 2020; Wu et al., 2021). Additionally, this cell line was shown to form long cilia similar to those in cone photoreceptor outer segments, making it useful for studying retinal ciliopathies, which include certain forms of RP and LCA (Wheway et al., 2019). In a recent study, increased expression of rod-specific genes was achieved in the 661W cells stably expressing the neural retinal leucine zipper (NRL) transcription factor. Using the resultant 661W-A11 cell line, an *in vitro* model of RP was created by inhibiting phosphodiesterase 6 (PDE6), and several neuroprotective drugs were subsequently tested using this model (Huang L. et al., 2021). Other well-known photoreceptor-like cell lines include the Y79 and WERI-RB1 cell lines derived from human retinoblastoma (Reid et al., 1974; McFall et al., 1977). The Y79 and WERI-RB1 cells originate from primitive multipotent retinoblasts; hence, they can partially differentiate into RPE cells as well as cells of neuronal or glial nature (Kyritsis et al., 1986). They express markers of various retinal cell types, including some markers of cone and rod photoreceptors (Bogenmann et al., 1988; Di Polo and Farber, 1995; Cassidy et al., 2012). Although the Y79 and WERI-RB1 cell lines are predominantly used for modeling retinal tumors *in vitro*, there are instances of their use in studying XLRS (Kitamura et al., 2011; Plössl et al., 2017).

The ARPE-19 cell line is a widely used RPE-like cell line derived from a primary culture of human RPE cells and initially characterized by pigmentation, expression of RPE-specific markers CRALBP and RPE65, and the ability to form polarized epithelial monolayers on porous filter substrates (Dunn et al., 1996). However, over time, this cell line changed, losing pigmentation and showing increased morphological heterogeneity depending on the maintenance conditions (Luo et al., 2006). Furthermore, the ARPE-19 cell line was not originally reported to be immortalized; the cells exhibited a tendency to senesce in culture (Dunn et al., 1996); however, later studies revealed that ARPE-19 subcultures contained subpopulations of both non-immortalized and immortalized cells (Kozlowski, 2015). For these reasons, there is currently a reevaluation of the suitability of ARPE-19 cells for basic, preclinical, and translational research (Pfeffer and Fliesler, 2022). Nonetheless, the ARPE-19 cell line continues to be actively used as an alternative to primary RPE cells to study the pathogenesis and drug testing for the treatment of various retinal diseases, including RP (Jiang et al., 2014; Hong et al., 2021), LCA, retinal ciliopathies (van Wijk et al., 2009; Nichols et al., 2010; Hidalgo-de-Quintana et al., 2015), and macular degenerations (Liao et al., 2019; Moine et al., 2021). Additionally, research is being conducted on culture conditions in which ARPE-19 cells can partially restore the functional and morphological phenotype characteristic of primary RPE cells. For instance, culturing in DMEM with the addition of pyruvate and glucose induces ARPE-19 cells to acquire pigmentation and express messenger RNAs (mRNAs), microRNAs (miRNAs), and certain proteins specific to RPE (RPE65, CRALBP, RDH5, RDH10, miR-204/211, etc.) (Ahmado et al., 2011; Samuel et al., 2017). Improved differentiation of ARPE-19 in a medium supplemented with nicotinamide (MEM-Nic) was also reported: cells acquired a cobblestone morphology and apical microvilli and expressed RPE-specific genes RPE65, BEST1, OCLN, MERTK, and ITGB5 (Hazim et al., 2019). Another study reported the creation of a pigmented ARPE-19mel cell line from ARPE-19 cells that spontaneously phagocytosed melanosomes isolated from pig RPE (Hellinen et al., 2019).

Another well-known RPE-like cell line, hTERT RPE-1, was created by immortalizing human RPE cells with human telomerase hTERT. The hTERT RPE-1 cells are capable of unlimited division but are not oncogenic (Jiang et al., 1999). This cell line is used to study retinal ciliopathies (Spalluto et al., 2013; Gómez et al., 2022), the interaction of RPE with Bruch’s membrane, and oxidative stress (Choudhury et al., 2021). For example, this cell line was used to test therapy for AD-RP caused by mutations in the NR2E3 gene based on antisense oligonucleotides (Naessens et al., 2019).

In addition to the cell lines mentioned above, non-retinal-specific cell lines, such as HEK293 (a cell line derived from human embryonic kidneys) and COS (a cell line derived from the kidney tissue of the African green monkey), can also be used for *in vitro* IRD modeling (Wang G. et al., 2009; Gopalakrishna et al., 2016; Sarkar et al., 2021, etc.).

### 2.2 Primary cells

Primary cell cultures of photoreceptors and RPE, isolated from the retinas of mice, pigs, or humans, exhibit the greatest similarity to *in vivo* retinal cells in terms of their function and morphology. However, obtaining such cultures is problematic and labor-intensive due to the small amount of starting material, low viability, and rapid dedifferentiation of cells post-isolation (Michelis et al., 2023). Creating *in vitro* models based on primary cells is further complicated by the irreproducibility of the isolation sources. Moreover, there is an added ethical issue with obtaining retinal cells from animals and humans due to the invasiveness of this procedure.

The use of primary photoreceptor cultures is limited by the short lifespan of isolated cells (a few days). Additionally, during retinal tissue dissociation (e.g., via enzymatic digestion with papain), the structural integrity of photoreceptor cells is generally compromised and their outer and inner segments and ribbon synapses are lost (Yang et al., 2001). Enhanced survival of primary photoreceptor cells can be achieved via treatment with neurotrophic factors such as basic fibroblast growth factor (FGF2) and epidermal growth factor (EGF) (Fontaine et al., 1998; Traverso et al., 2003; Forouzanfar et al., 2020). Furthermore, cultivating photoreceptor cells in media conditioned by retinal Müller glial cells, which secrete glial cell line-derived neurotrophic factor (GDNF) (Del Río et al., 2011), insulin-like growth factor-binding protein (IGFBP5), and connective tissue growth factor (CTGF) (Hauck et al., 2008), also promotes cell longevity.

The high purity of photoreceptor populations essential for both cell therapy and disease modeling can be achieved by fluorescence-activated cell sorting (FACS) (Lakowski et al., 2018), magnetic-activated cell sorting (MACS) (Eberle et al., 2011), and real-time deformability cytometry (RT-DC) (Santos-Ferreira et al., 2019). For instance, rod cultures are often isolated with the help of antibodies against CD73, a surface marker of common cone/rod precursors and mature rod cells (Koso et al., 2009). Additional methods help isolate specific photoreceptor types, such as cone-only or rod-only cultures; for example, cone cultures can be isolated using the ability of these cells to specifically bind to peanut agglutinin lectin (PNA) (Balse et al., 2005; Skaper, 2012).

Primary RPE cells are also challenging to isolate: extraction from their native environment leads to loss of pigmentation, dedifferentiation, and the acquisition of a mesenchymal phenotype (Klettner, 2020). However, numerous protocols have been developed to mitigate these issues to varying degrees of success (Fronk and Vargis, 2016). For example, since the epithelial-mesenchymal transition (EMT) of RPE cells is triggered by the loss of tight intercellular junctions, culturing human RPE as fragments of the cell layer isolated from the eyes has been proposed (Blenkinsop et al., 2013). Additionally, it was reported that incubation in media with increased calcium content or with the Rac1 inhibitor, a regulatory factor associated with cell migration, promotes the formation of tight junctions and uniform maturation of human RPE cells (McKay and Burke, 1994; Rak et al., 2006; Sonoi et al., 2016). In another study, EMT in mouse RPE cells was prevented by the addition of Y27632 and Repsox, inhibitors of Rho-kinase and TGFβR-1/ALK5, respectively (Shen et al., 2017). Recent research focused on the impact of various coatings and carrier materials on primary RPE cell proliferation, differentiation, and function (Tichotová et al., 2022; Dörschmann et al., 2022). At present, cells are most frequently cultured on Transwell membranes (Fernandez-Godino et al., 2016; Hood et al., 2022). For example, primary porcine (Pilgrim et al., 2017) and human (Rabin et al., 2011) RPE cells cultured on Transwell membranes have been used to study subretinal deposit formation in early AMD. In addition, primary RPE cells can be cultured on materials that mimic Bruch’s membrane, such as collagen, fibronectin, Matrigel, and others; this approach enhances their functionality as an *in vitro* model of AMD and other retinal diseases (Murphy et al., 2020).

### 2.3 Stem cells

Multipotent and pluripotent stem cells are undifferentiated cells capable of self-renewal and differentiation into specific cell types. Multipotent cells can differentiate into a limited number of cell types, typically those of a particular tissue, while pluripotent cells possess a greater differentiation potential and can differentiate into all cell types of an adult organism (Tian et al., 2023). *In vitro* IRD models rely on both multipotent and pluripotent stem cells and their ability to differentiate into photoreceptor or RPE cells (Achberger et al., 2019). Several strategies for controlled differentiation into retinal cells have been developed. The most common approach involves culturing cells in media containing various growth factors and compounds responsible for activation or inhibition of specific cellular signaling pathways (Huang Y. et al., 2018; Kadkhodaeian et al., 2019b; Afanasyeva et al., 2021, among others). Another method of differentiation relies on co-culturing with retinal cells (usually primary cells or RPE cell lines) that secrete factors promoting differentiation into the media (Duan et al., 2013; Zhang Y. et al., 2017, among others). Additionally, the expression of transcription factors in differentiating cells can be achieved by either viral transduction or transfection of cells with antisense miRNAs to mature miRNAs, which inhibit genes relevant to retinal development (Yan et al., 2013; Choi S. W. et al., 2016; Zhu X. et al., 2022).

#### 2.3.1 Multipotent stem cells

Multipotent stem cells can differentiate into specific types of retinal cells and include such cells as fetal neural lineage stem cells (e.g., retinal progenitor cells), adult neural lineage stem cells (e.g., retinal cells capable of differentiation), and adult non-neural lineage stem cells (e.g., mesenchymal stem cells) (Canto-Soler et al., 2016). Compared to adult stem cells, fetal stem cells exhibit higher self-renewal and differentiation capacities; however, their use is associated with ethical concerns. Consequently, fetal neural stem cells are rarely used.

Retinal cells capable of differentiation include retinal ciliary epithelial stem cells, Müller glial cells, and RPE stem cells (Jeon and Oh, 2015). Retinal ciliary epithelial stem cells (CESC) are a small population of cells in the human eye that demonstrate proliferation, self-renewal capacity, and multipotency after isolation (Coles et al., 2004). A number of studies confirmed that CESCs are capable of differentiating into retinal ganglion cells and rod photoreceptors (Das A. V. et al., 2005; Ballios et al., 2012; Del Debbio et al., 2013). However, over time, the differentiation potential of these cells and their stemness have been questioned (Frøen et al., 2013). Müller glial cells are not typical stem cells but have significant potential to differentiate into retinal cells (Gao et al., 2021), retinal ganglion cells, and rod photoreceptors (Giannelli et al., 2011; Singhal et al., 2012; Zeng et al., 2023). RPE stem cells (RPESC), a subpopulation of RPE cells, are capable of self-renewal, proliferation, dedifferentiation with loss of RPE markers, and differentiation into retinal nerve cells and cells of the mesenchymal lineage under certain conditions (Salero et al., 2012; Saini et al., 2016). RPESCs are considered a source of RPE cells with characteristics of native RPE for cell replacement therapy (Blenkinsop et al., 2015). In addition, RPE cells are amenable to reprogramming into photoreceptor cells induced by overexpression of NeuroD, Ngn1, and Ngn3 (neurogenin 1 and 3) proteins (Yan et al., 2013). In addition to the above-mentioned cells of the retina, the human eye contains other stem cells, some of which are capable of differentiating into retinal cells due to the common ancestry. And finally, chick and pig iris stroma and pig iris pigment epithelial cells also have the capacity to differentiate into neuronal and photoreceptor-like cells *in vitro* (Matsushita et al., 2014; Royall et al., 2017). At present, the described cell types are more commonly used in regenerative medicine applications than in IRD modeling (Xiao et al., 2024).

#### 2.3.2 Pluripotent stem cells

Pluripotent stem cells include ESCs and iPSCs derived by reprogramming adult somatic cells such as fibroblasts, keratinocytes, and peripheral blood mononuclear cells (PBMCs). Unlike multipotent cells, PSCs can be differentiated into both individual retinal cell types and ROs containing all major retinal cell types and mimic the structure of the retina *in vivo* (Afanasyeva et al., 2021). For example, 2D and 3D iPSC-derived *in vitro* models already exist for such diseases as RP, LCA, SD, BVMD, and choroideremia (Seah et al., 2024). ROs hold great promise for studying retinogenesis and healthy retinal physiology, modeling retinal diseases *in vitro*, and for cell replacement therapy (Cheng and Kuehn, 2023; Liang et al., 2023; Kurzawa-Akanbi et al., 2024). There are various protocols for creating ROs; they rely on different cell sources, reprogramming approaches, and differentiation methods. These protocols, as well as other features of organoids and their characteristics, have been described in detail in a number of reviews. In recent years, the technology for generating ROs has been actively combined with the latest organ-on-chip and 3D bioprinting technologies, which is expected to enhance the functionality of organoids and advance their use in the aforementioned fields (Zhao and Yan, 2024). However, to date, the production of ROs has been characterized by a number of difficulties: high costs, long cultivation time to achieve differentiation into mature and functional photoreceptors, low differentiation yield, and the high heterogeneity of organoid cultures obtained under different conditions (Li X. et al., 2021). The use of PSCs is also associated with a number of difficulties. For instance, the use of ESCs is characterized by ethical problems and, as a consequence, low availability (Volarevic et al., 2018), while iPSCs are genetically unstable and are known for epigenetic changes, which limits their application (Kim K. et al., 2010; Polo et al., 2010; Yoshihara et al., 2019). These factors create a risk of oncogenesis and make it difficult to control the directed differentiation of iPSCs. Despite this, the development of *in vitro* models of retinal diseases based on PSCs, in particular iPSCs, is a promising direction, since ROs derived from these cells reproduce *in vivo* conditions most reliably among existing cellular models. Still, iPSCs are not able to fully replace the existing models due to laborious protocols, the high cost of obtaining them, the low yield of long-term differentiation, the high heterogeneity of the obtained structures, and ethical and practical problems with cell sources.

### 2.4 Somatic cells

It is also noteworthy that certain somatic cells can be directly reprogrammed into retinal cells with varying degrees of efficiency. For instance, iris pigment epithelial cells, fibroblasts, and human PBMCs have all been reprogrammed into photoreceptor-like retinal cells (Seko et al., 2012; Seko et al., 2014; Komuta et al., 2016). This reprogramming was achieved by retrovirus- or Sendai virus-mediated delivery of transcription factors CRX, RX, NEUROD, and OTX2 in various combinations. Furthermore, to create an *in vitro* model of a type of RP, fibroblasts derived from patients with mutations in the EYS gene were isolated and transduced with the aforementioned transcription factors using a retroviral vector (Seko et al., 2018; Rai et al., 2022). Another study reported chemically induced reprogramming of fibroblasts into rod-like photoreceptor cells using VCRF, a combination of valproic acid, CHIR99021 (a GSK3 inhibitor), repsox, and forskolin; STR, a combination of Sonic hedgehog (Shh), taurine, and retinoic acid; and IWR1 (a Wnt/β-catenin pathway inhibitor) (Mahato et al., 2020). Additionally, human fibroblasts can be reprogrammed into stable RPE-like cells via lentivirus-delivered transcription factors MITF, OTX2, LIN28, MYC, and CRX (Woogeng et al., 2022).

Overall, direct reprogramming of somatic cells is a simpler and cheaper method for generating retinal cells compared to the stem cell-derived approaches. However, this method has a major drawback: significantly reduced differentiation efficiency, as evidenced by the low expression levels of retinal cell markers and morphological discrepancies.

## 3 Mesenchymal stem cells as a potential optimal source for creating in vitro models of IRDs

### 3.1 Characteristics of MSCs

**FIGURE 3:**
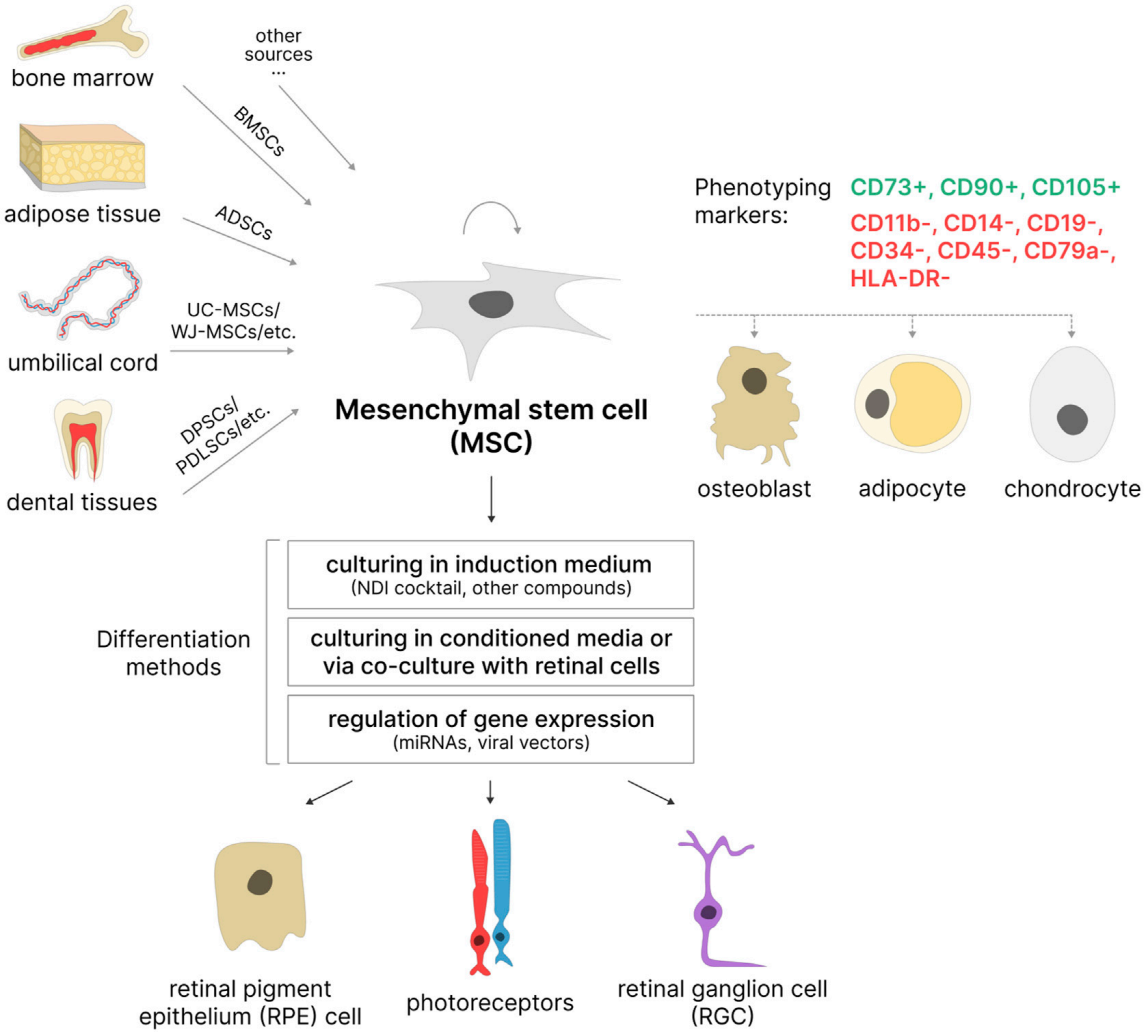
MSCs as a potential source of retinal cells. MSCs can be isolated from various sources based on phenotyping markers. Most often used: BMSCs–bone marrow stem cells, ADSCs–adipose-derived stem cells, UC-MSCs–umbilical cord MSCs, WJ-MSCs–Wharton’s jelly MSCs, DPSCs–dental pulp stem cells; PDLSCs–periodontal ligament stem cells, etc. These cells are capable of self-renewal and differentiation in three directions: osteogenic, adipogenic, and chondrogenic. Under certain conditions, they are able to differentiate into retinal pigment epithelial (RPE) cells, photoreceptors, and retinal ganglion cells (RGCs).

Mesenchymal stem cells are adult multipotent stem cells with fibroblast-like morphology, high self-renewal capacity, and multilineage differentiation potential. The International Society for Cellular Therapy (ISCT) formulated minimal criteria for human MSCs: i) the cells must be capable of adhering to plastic surfaces; ii) they must express the surface markers CD105, CD73, and CD90 while lacking expression of hematopoietic markers CD45, CD34, CD14 or CD11b, CD79a or CD19, and HLA-DR; and iii) they must be capable of differentiation into osteoblasts, adipocytes, and chondrocytes *in vitro* (Dominici et al., 2006) (Figure 3). It is important to note that ISCT criteria apply only to MSCs cultured *in vitro* and may not fully reflect the properties of MSCs *in vivo* (McNiece, 2007). Furthermore, MSCs isolated according to these criteria often represent a phenotypically heterogeneous population. Consequently, additional cell surface markers, such as CD271, CD106, and CD146, are sometimes used to isolate subpopulations of MSCs with high proliferative capacity and greater multilineage differentiation potential (Mo et al., 2016). The exact characteristics of MSCs (including the ability to differentiate into multiple lineages) vary depending on the isolation source, donor age, isolation method, and composition of the cell culture medium (Mushahary et al., 2018; Andrzejewska et al., 2019). Moreover, MSCs obtained from donors of the same age may also differ in their proliferative and differentiation potential (Li J. et al., 2023).

Additionally, MSCs possess the ability to secrete neurotrophic, immunosuppressive, and anti-angiogenic factors, which enhances their application in cell replacement therapies for various diseases, including degenerative retinal diseases (Adak et al., 2021).

### 3.2 Sources of MSCs

Bone marrow-derived MSCs (BMSCs) and adipose-derived stem cells (ADSCs) are the most commonly used among adult MSC sources. BMSCs were among the first described MSCs (Friedenstein et al., 1987) and are still considered a promising cell source due to their high colony-forming potential and multilineage differentiation capacity *in vitro* (Chu et al., 2020; Purwaningrum et al., 2021). However, bone marrow aspiration is an invasive procedure associated with significant pain, which is a considerable drawback of this method. ADSCs have somewhat similar morphology to BMSCs, comparable proliferative ability, and high differentiation potential (Strioga et al., 2012; Bunnell, 2021). Moreover, ADSCs are typically obtained from biological material collected through the less-invasive procedure of liposuction, making them more accessible than BMSCs. MSCs derived from dental tissues, such as dental pulp stem cells (DPSCs), stem cells from human exfoliated deciduous teeth (SHED), and periodontal ligament stem cells (PDLSCs), can be used for neuronal differentiation (Aydin and Şahin, 2019; Sramkó et al., 2023). Teeth can be obtained without ethical concerns as they are considered biological waste in dentistry (Das and Sloan, 2023). Among neonatal sources, the umbilical cord is particularly popular: MSCs can be isolated from the whole umbilical cord (UC-MSCs), Wharton’s jelly (WJ-MSCs), or umbilical cord blood (UCB-MSCs) (Mennan et al., 2013; Nagamura-Inoue and He, 2014). The umbilical cord is considered medical waste, making it an accessible source that does not require invasive procedures for MSC isolation (Das and Sloan, 2023). Additionally, umbilical cord MSCs have further advantages: an increased proliferative potential and a higher number of cell passages *in vitro* before reaching senescence compared to adult MSCs (Hass et al., 2011). It is also worth noting that umbilical cord tissues are richer sources of MSCs compared to umbilical cord blood (Jin et al., 2013), and cells isolated from the whole umbilical cord are easier to obtain, proliferate faster, and are more durable in culture than those from Wharton’s jelly (Mennan et al., 2016).

Overall, the choice of MSC source depends on the intended application, immunomodulatory properties, ability to secrete specific factors, proliferative capacity, and differentiation potential in certain directions.

### 3.3 Differentiation of MSCs into osteogenic, adipogenic, and chondrogenic lineages

MSCs are capable of differentiating into mesodermal lineage cells, and the characterization of isolated MSCs requires confirmation of their ability to differentiate into osteoblasts, adipocytes, and chondrocytes *in vitro*.

Osteogenic differentiation is commonly initiated in a medium supplemented with dexamethasone, β-glycerophosphate, and ascorbic acid (Pittenger et al., 1999). The success of differentiation is assessed by the increased levels of expression of alkaline phosphatase and the formation of calcium deposits, which can be visualized with the help of specific stains such as Alizarin Red S (Ciuffreda et al., 2016).

Adipogenic differentiation is typically induced in a medium containing dexamethasone, insulin, indomethacin, ascorbic acid, and other compounds (Rosen and MacDougald, 2006; Scott et al., 2011). Confirmation of adipogenic differentiation is carried out by staining lipid droplets with specific dyes, such as Oil Red O (Ciuffreda et al., 2016).

Chondrogenic differentiation is carried out in media supplemented with dexamethasone, ITS (insulin-transferrin-selenium), ascorbic acid, pyruvate, TGF-β1, and other compounds (Solchaga et al., 2011; Narcisi et al., 2021). The differentiation results in the formation of cell spheres that express type II collagen, which can also be stained with dyes such as Alcian Blue (Ciuffreda et al., 2016).

Additionally, the ability of MSCs to differentiate into these lineages can be confirmed by assessing the expression levels of genes specific to osteocytes, adipocytes, and chondrocytes.

### 3.4 Differentiation of MSCs into retinal cells

Under specific conditions, MSCs can differentiate into cells of ectodermal (e.g., neurons, epithelial cells) or endodermal (e.g., hepatocytes) origin (Sierra-Sánchez et al., 2018; Hernández et al., 2020; Afshari et al., 2020). The ability of various MSCs to efficiently differentiate into retinal cells *in vitro* can be utilized in both cell therapy and the development of cellular models for retinal diseases.

As previously mentioned, several strategies exist for differentiating stem cells into retinal cells *in vitro*. These strategies aim to mimic the *in vivo* conditions of retinal maturation at different stages by activating or inhibiting specific cellular signaling pathways. The differentiation factors can be either added to the culture medium or are secreted by retinal cells during co-culture. Additionally, some differentiation protocols rely on regulation of the expression of certain genes involved in this process. Supplementary Table S1 provides detailed data on existing methods of differentiation of MSCs into retinal cells.

#### 3.4.1 Differentiation in induction media

Currently, the differentiation of MSCs into retinal neurons (including photoreceptors) often relies on the neural differentiation induction NDI cocktail, which includes Noggin (a bone morphogenetic protein BMP pathway inhibitor), Dickkopf-1 (a Wnt/β-catenin pathway inhibitor), and IGF1 (insulin-like growth factor 1). Noggin inhibits the BMP pathway, promoting neural tube patterning and the differentiation of cells into retinal neurons (McMahon et al., 1998; Lan et al., 2009; Tao et al., 2010). Inhibition of the Wnt/β-catenin pathway via Dickkopf-1 (Dkk-1) promotes the differentiation of retinal progenitor cells into retinal neurons (Das A. V. et al., 2008). IGF1 is crucial for anterior neural system development and supports the maturation of retinal progenitor cells into photoreceptor cells (Pera et al., 2001; Wang Y. et al., 2018; Zerti et al., 2021).

Enhancing differentiation efficiency can also involve inhibition of the Notch1 pathway, for example, with DAPT (N-[N-(3, 5-diflurophenylacetate)-L-alanyl]-(S)-phenylglycine t-butyl ester), a γ-secretase inhibitor. Active Notch1 signaling is associated with the proliferation of retinal progenitor cells, while its inhibition promotes their differentiation into photoreceptors (Jadhav et al., 2006; Mills and Goldman, 2017). Other compounds, such as Shh, triiodothyronine (T3), trans-retinoic acid, and taurine, are also used for induction of retinal neuron differentiation. Shh plays a role in neuroretina development and visual field formation, promoting the differentiation of retinal progenitors into rod photoreceptors (Levine et al., 1997). T3 determines cone subtypes by suppressing the formation of S-cones and inducing the formation of L/M-cones (Eldred et al., 2018). Trans-retinoic acid and taurine stimulate rod photoreceptor development (Altshuler et al., 1993; Kelley et al., 1994; Khanna et al., 2006; Khalili et al., 2018).

Differentiation is often carried out in DMEM/F12-based media supplemented with B27 and N2 for cultivating neuronal cells, ITS, and neurotrophic factors such as FGF2, EGF, BDNF (brain-derived neurotrophic factor), CNTF (ciliary neurotrophic factor), NGF (nerve growth factor), and others. The most common protocol involves culturing MSCs under ultra-low adherence conditions to differentiate neurons, followed by culturing the resulting neurospheres under adhesive conditions (see protocols in Supplementary Table S1).

Human PDLSCs were differentiated into retinal cells via the formation of neurospheres under low-attachment conditions, followed by adherent culture in media containing B27, N2, Noggin, and Dkk-1. The differentiated cells exhibited increased expression of retinal progenitor (LHX2, DCX, CHX10, RX, SOX2, OTX2) and photoreceptor (NRL, RHO) genes (Huang L. et al., 2013). Later, PDLSCs were differentiated into RGC using a modified protocol that included IGF1, FGF2, ITS, BDNF, CNTF, NGF, and Shh in the medium. As a result, the cells expressed markers of retinal progenitors (PAX6, CHX10) and retinal ganglion and neuronal cells (TUBB3, MAP2, TAU, NEUROD1, SIX3, ATOH7, and POU4F2) (Ng et al., 2015).

Following a similar protocol, differentiated ADSCs exhibited expression of marker genes for retinal progenitor cells (PAX6 and NES), photoreceptors and their precursors (CRX, NRL, RHO, and RCVRN), and RGC (ATOH7, TUBB3, and POU4F2). Additionally, the impact of the Notch1 pathway on ADSC differentiation was investigated: activation of the pathway with JAG1 enhanced the expression of retinal progenitor markers, while inhibition with DAPT led to increased expression of RGC genes (Huang Y. et al., 2018). In a subsequent study, ADSC differentiation into photoreceptors and RGC was compared under adherent and non-adherent conditions. It was found that ultra-low attachment conditions could facilitate differentiation into the retinal lineage; however, the combination of the NDI differentiation cocktail and non-adherent culture conditions did not enhance differentiation efficiency (Ling et al., 2023).

Rat DPSCs were differentiated into RGC in a medium containing N2, heparin, FGF2, and Shh in both 2D and 3D cultures within fibrin hydrogel, mimicking the mechanical properties of the developing retina. Enhanced expression of retinal neuronal and ganglion cell markers (PAX6, ATOH7, MAP2, POU4F2, and GFAP) was observed in the 3D culture compared to the 2D culture (Roozafzoon et al., 2015). In another study, SHED were successfully differentiated into retinal photoreceptor cells in a medium containing the NDI differentiation cocktail, along with B27, N2, ITS, FGF2, Shh, T3, and trans-retinoic acid. Differentiated cells demonstrated expression of neuronal markers (NEUROD1, ASCL1, TAU, GluR2, OTX2, and AIPL1), retinal progenitor markers (PAX6, RX, and CHX10), photoreceptor precursor markers (RCVRN, CRX, and NRL), and photoreceptor markers (RHO and OPN1SW) at different stages (Li X. et al., 2019).

Additionally, differentiation of MSCs derived from human olfactory mucosa (OM-MSCs) into retinal photoreceptor cells in the presence of EGF, taurine, and retinoic acid in the medium was confirmed by the expression of the rod photoreceptor marker RHO (Lu et al., 2017). Taurine was also used to induce the differentiation of conjunctiva mesenchymal stem cells (CJMSCs) cultured on poly-l-lactic acid (PLLA) nanofibrous scaffolds, which led to their differentiation into photoreceptor-like cells and the expression of rod photoreceptor markers (CRX, RCVRN, and RHO). Moreover, the expression of these genes was higher in cells cultured on randomly-oriented scaffold nanofibers than on aligned scaffold nanofibers (Nadri et al., 2013). Later, the same authors demonstrated the differentiation of CJMSCs into photoreceptor-like cells on scaffolds made of polycaprolactone (PCL) and polyethylene glycol (PEG) with the addition of taurine; the resulting cells expressed some photoreceptor markers (RCVRN, RHO) (Nadri et al., 2017).

Differentiation of stem cells into RPE cells can also be achieved in media containing inhibitors of the BMP and Wnt/β-catenin signaling pathways. The efficiency of differentiation into RPE cells can be increased by the addition of nicotinamide, activin A, and other compounds to the medium (Idelson et al., 2009; Hazim et al., 2019). Media is supplemented with EGF, FGF2, ITS, and similar factors for the maintainance of differentiating cells (see protocols in Supplementary Table S1). For example, rat BMSCs were differentiated into pigmented spheres capable of forming monolayers of RPE-like cells with phagocytic activity towards photoreceptor outer segments (POS). The differentiation medium contained EGF, FGF2, insulin, T3, putrescine, selenium, and linoleic acids. Initially, neurospheres expressed stem cell markers (OCT4, SOX2, NANOG) and neural stem cell marker (NES). Subsequently, pigmented spheres expressed retinal progenitor marker OTX2 required for RPE specification and RPE markers RPE65 and CRALBP (Kadkhodaeian et al., 2019b). In another study by these authors, ADSCs were differentiated into RPE cells in induction medium containing insulin, T3, and EGF. After 80 days of differentiation, cells expressed RPE markers (RPE65 and CRALBP) and exhibited epithelial morphology (Kadkhodaeian et al., 2019a).

#### 3.4.2 Differentiation in conditioned media or via co-culture with retinal cells

Differentiation of MSCs into RPE cells is frequently performed using conditioned media or by co-culturing MSCs with primary RPE cells or RPE cell lines, such as ARPE-19, due to the simplicity and relative cost-effectiveness of this approach (see protocols in Supplementary Table S1).

For example, human ADSCs were differentiated into RPE-like cells in media conditioned by primary porcine or human RPE cells and/or containing vasoactive intestinal peptide (VIP). Following differentiation, these cells expressed RPE markers (bestrophin, RPE65, and CK8/18) and produced melanin pigment in response to hormonal stimulation by melanocyte-stimulating hormone. Interestingly, the combined use of conditioned media and VIP did not enhance the differentiation (Vossmerbaeumer et al., 2009). In another study, ADSCs were cultured in ARPE-19-conditioned media, resulting in cells expressing RPE markers (bestrophin, RPE65, and CK8) and exhibiting increased proliferative and migratory capabilities (Zhang Y. et al., 2017).

Additionally, RPE-like cells were generated by co-culturing human BMSCs with porcine RPE cells in a Transwell system. The differentiated cells expressed markers of RPE and their progenitors (MITF, OTX2, bestrophin, tyrosinase, PMEL17, RPE65, ZO-1, PEDF, and CRALBP), displayed the presence of pigmented granules, phagocytosed POS, and secreted BDNF and GDNF (Duan et al., 2013). Similarly, co-culturing ARPE-19 cells with WJ-MSC in a Transwell system resulted in expression of RPE-specific markers (MITF, OTX2, RPE65, PEDF, PMEL17, CRALBP, and ZO-1), phagocytic ability, and secretion of BDNF and GDNF (Chang et al., 2022).

Co-cultivation can be achieved without the use of Transwell systems when MSCs are co-cultured together with UV-inactivated RPE cells. For instance, rabbit BMSCs were differentiated into RPE-like cells via co-cultivation with UV-inactivated ARPE-19 cells in a gellan gum-based hydrogel supplemented with B27. The differentiated cells expressed RPE-specific markers (RPE65, NPR-A, and CRALBP) (Choi M. J. et al., 2019).

In addition to differentiation into RPE-like cells, the co-cultivation approach with RPE cells can be used for differentiation into photoreceptor-like cells. For example, BMSCs were initially cultured in a neurogenic differentiation medium containing FGF2, EGF, ITS, and other compounds, followed by incubation in a medium with UV-inactivated human RPE cells. Neurospheres obtained from neurogenic differentiation expressed the marker of neural precursor cells, NES, while photoreceptor-like cells obtained from further differentiation expressed markers associated with photoreceptors (PKC and opsins) (Chiou et al., 2005).

#### 3.4.3 Differentiation via regulation of gene expression

Regulation of specific gene expression in cells is often achieved by retroviral and lentiviral transduction, which offers the advantage of long-term and stable gene expression (see protocols in Supplementary Table S1). For instance, in ADSCs, the transcription factor PAX6 (5a) was stably expressed post-lentiviral transduction, followed by culturing the transduced cells in a fibronectin-containing medium. As a result, the cells expressed markers of retinal precursor cells (PAX6, CHX10), RPE markers (RPE65, CRALBP, CK8/18), and photoreceptor cells and their precursors (CRX, NRL, RCVRN, and RHO) (Rezanejad et al., 2014). In another study, UC-MSCs were differentiated through retroviral delivery of transcription factors CRX, NR2E1, C-MYC, LHX2, and SIX6. The resulting RPE-like cells expressed RPE markers (RPE65, MERTK, TYRP1, CRALBP, PEDF, and ZO-1), exhibited phagocytic ability towards POS, and possessed characteristics similar to those of RPE cells derived from iPSCs (Zhu X. et al., 2022).

Another method of regulation of gene expression involves the use of anti-sense miRNAs to mature miRNAs for inhibition of their expression via RNA interference (see protocols in Supplementary Table S1). miRNAs in cells regulate gene expression by binding to target mRNAs, leading to their degradation or inhibition of translation. A single miRNA can target the expression of multiple genes. MiRNAs play a crucial role in the differentiation process and maintenance of the undifferentiated state (Guo et al., 2011). Several studies describe the differentiation of human amniotic epithelial SCs (AESCs) and UCB-MSCs by inhibiting cell miRNAs. For example, inhibition of miRNA410, targeting OTX2 and RPE65 genes, promoted the differentiation of cells into RPE-like cells expressing RPE markers (MITF, RPE65, bestrophin, EMMPRIN, etc.) and exhibiting phagocytic activity (Choi S. W. et al., 2015; Choi S. W. et al., 2017). Inhibition of miRNA203, targeting DKK1, CRX, NRL, NEUROD1, and RORβ genes, led to differentiation into photoreceptor-like cells expressing markers of rod and cone photoreceptors and their precursors (THRB, NR2E3, NRL, OPN1MW, etc.) (Choi S. W. et al., 2016).

Some studies also described CJMSCs transduced with lentivirus delivering let-7a miRNA, which is normally expressed in neural stem cells during retinal retinogenesis. CJMSCs overexpressing let-7a miRNA differentiated into photoreceptor-like cells and expressed rod photoreceptor markers (RCVRN, RHO) (Ranjbarnejad et al., 2019). In addition, CJMSCs were transduced with lentiviruses delivering miRNA-9, which also plays a role in retinal development. In one study, CJMSCs overexpressing miRNA-9 were cultured on a silk fibroin-poly-L-lactic acid (SF-PLLA) scaffold and differentiated into photoreceptor-like cells expressing rod photoreceptor markers (RCVRN, RHO) (Rahmani et al., 2020). In another study, CJMSCs overexpressing miRNA-9 were cultured on the scaffold obtained by polymerization of silk fibroin and reduced graphene oxide nanoparticles (SF-rGo) and electrically induced to differentiate. The resulting cells expressed photoreceptor markers (PRPH, RCVRN, and RHO) (Naderi and Nadri, 2022).

### 3.5 Genetic engineering of MSCs

**FIGURE 4:**
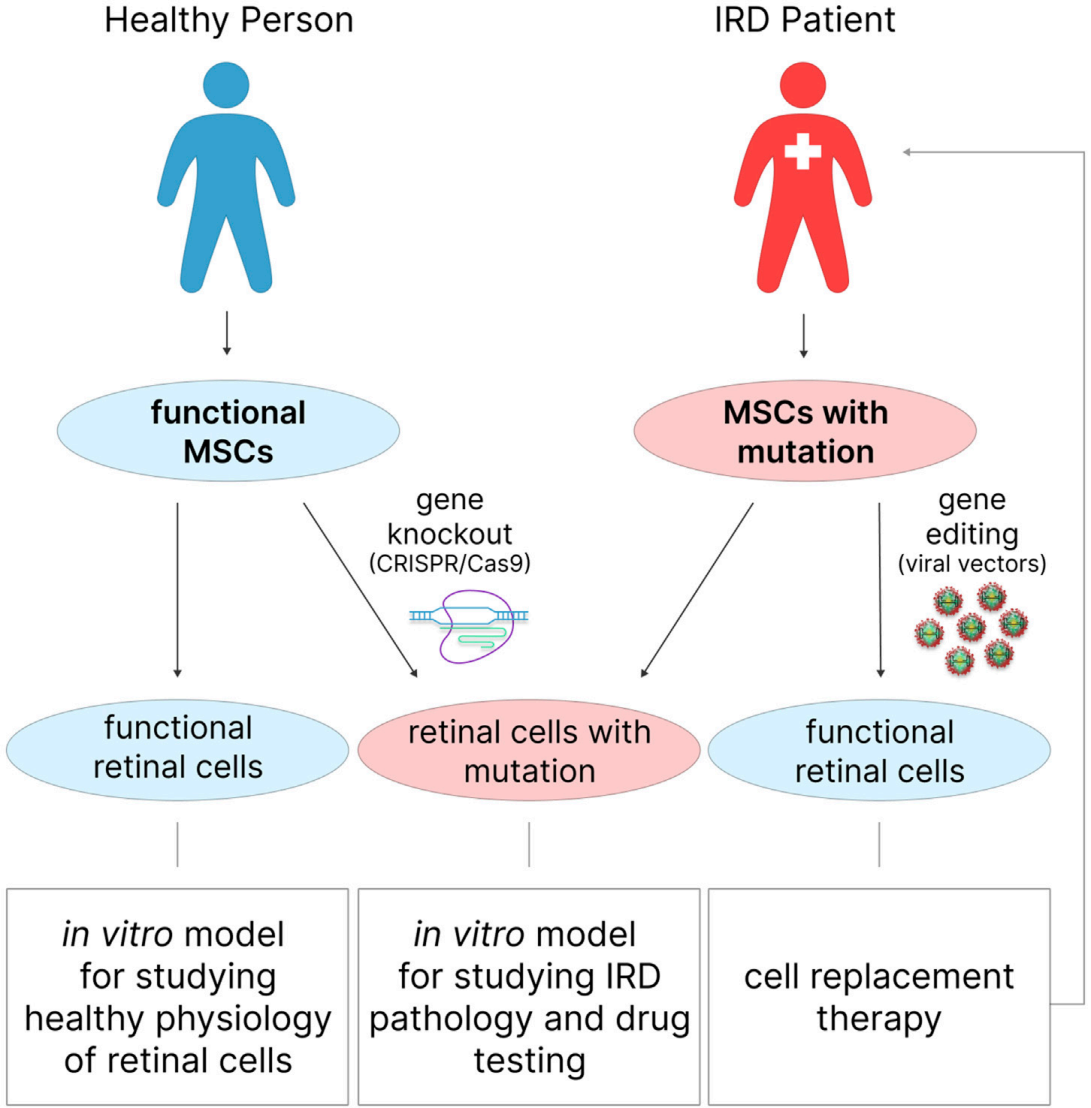
MSCs for *in vitro* modeling of healthy retinal physiology, or IRDs, and in cell therapy of degenerative retinal diseases. Functional retinal cells can be obtained from the MSCs of a healthy donor and are suitable for studying healthy physiology. Similar to retinal cells obtained from IRD patient MSCs, gene-edited MSCs from healthy donors can be used to study IRD pathology or for drug testing.

The development of *in vitro* IRD models relies on the ability to genetically edit cells in order to introduce disease-causing pathogenic mutations. It is advisable to use the so-called gene-cell approach, which involves the initial genetic editing of cells followed by their differentiation into retinal cells using the methods described above. This step is necessary if the initial MSCs are isolated from a healthy donor rather than from an IRD donor (Figure 4). In the latter case, after differentiation into retinal cells, MSCs obtained from an IRD patient will be able to reproduce the corresponding genetic and cellular context of the disease without additional manipulations, which can be particularly useful for studying the pathological processes underlying it.

Gene knockout, often relying on CRISPR/Cas9 technology, is performed to reproduce the pathological state of cells resulting from mutations in specific genes and, consequently, the disruption of the function of specific proteins (Nie and Hashino, 2017). Compared to other gene engineering technologies such as zinc finger nucleases (ZFNs) and transcription activator-like effector nucleases (TALENs), CRISPR/Cas9 technology is more cost-effective, straightforward, efficient, and can be applied to editing multiple gene targets (Boti et al., 2023). This approach involves the synthesis of complementary guide RNA (gRNA) targeting a specific DNA locus to direct the Cas9 endonuclease for double-strand DNA cleavage (Ran et al., 2013). Reparation of such a break can occur via non-homologous end joining (NHEJ) or homology-directed repair (HDR). NHEJ leads to gene knockout due to the deletion and reading frame shift of the target DNA sequence. For HDR to occur, the presence of a homologous sequence is required, resulting in either the correction of the existing gene sequence or the insertion of the gene of interest. Often, during HDR, the green fluorescent protein (GFP) gene is inserted as a reporter for knockout cell selection (Ebrahim et al., 2020).

Gene editing of MSCs can be applied not only to the development of *in vitro* models but also to enhancing the therapeutic potential of MSCs in regenerative medicine by altering the secretion of various cytokines, growth factors, and other compounds in order to improve stemness characteristics, aging, migration, proliferation, anti-inflammatory properties, etc. (Damasceno et al., 2020; Hazrati et al., 2022). Viral (retro-, lenti-, and adeno-associated viral vectors) and non-viral (electroporation, microinjection, the use of chemical carriers, etc.) gene delivery systems are actively employed for these purposes (Ebrahim et al., 2020). The choice of gene delivery method for MSC modification depends on the properties of MSCs isolated from a specific source and the ultimate goals of application. Generally, viral vectors are considered the most promising gene delivery methods for MSCs as they are efficient and capable of ensuring long-term gene expression.

In some cases, genetic editing of MSCs is carried out to enhance their differentiation potential in a specific direction. For example, expression of the erythropoietin (EPO) gene delivered by lentiviral vector into WJ-MSCs led to more efficient differentiation of the cells into rod photoreceptors in the presence of taurine compared to non-transduced cells (Ding et al., 2019). Studies modifying MSCs to enhance osteogenic potential (Freitas et al., 2021) and chondrogenic potential (Kim H. J. et al., 2022) have also been published.

### 3.6 Use of MSCs in clinical trials

Currently, the use of MSCs in cell therapy for degenerative retinal diseases is quite promising. Their therapeutic effect can be attributed to three main properties: a paracrine protective effect on retinal cells, immunosuppressive properties, and the ability to differentiate into retinal cells in the appropriate cellular environment. However, the effect of MSC-based therapy is largely associated with neuroprotection of retinal cells and suppression of the inflammatory response, which helps slow or halt the progression of retinal degeneration (Chen et al., 2022). In addition, MSCs exhibit pro- or anti-angiogenic properties depending on the tissue microenvironment. The anti-angiogenic properties of MSCs are beneficial for the treatment of retinal diseases with pathological angiogenesis, such as AMD, diabetic retinopathy, and others. At the same time, their pro-angiogenic properties, useful in the restoration of ischemic damage of the retina, can contribute to the progression of these diseases (Adak et al., 2021).

**FIGURE 5:**
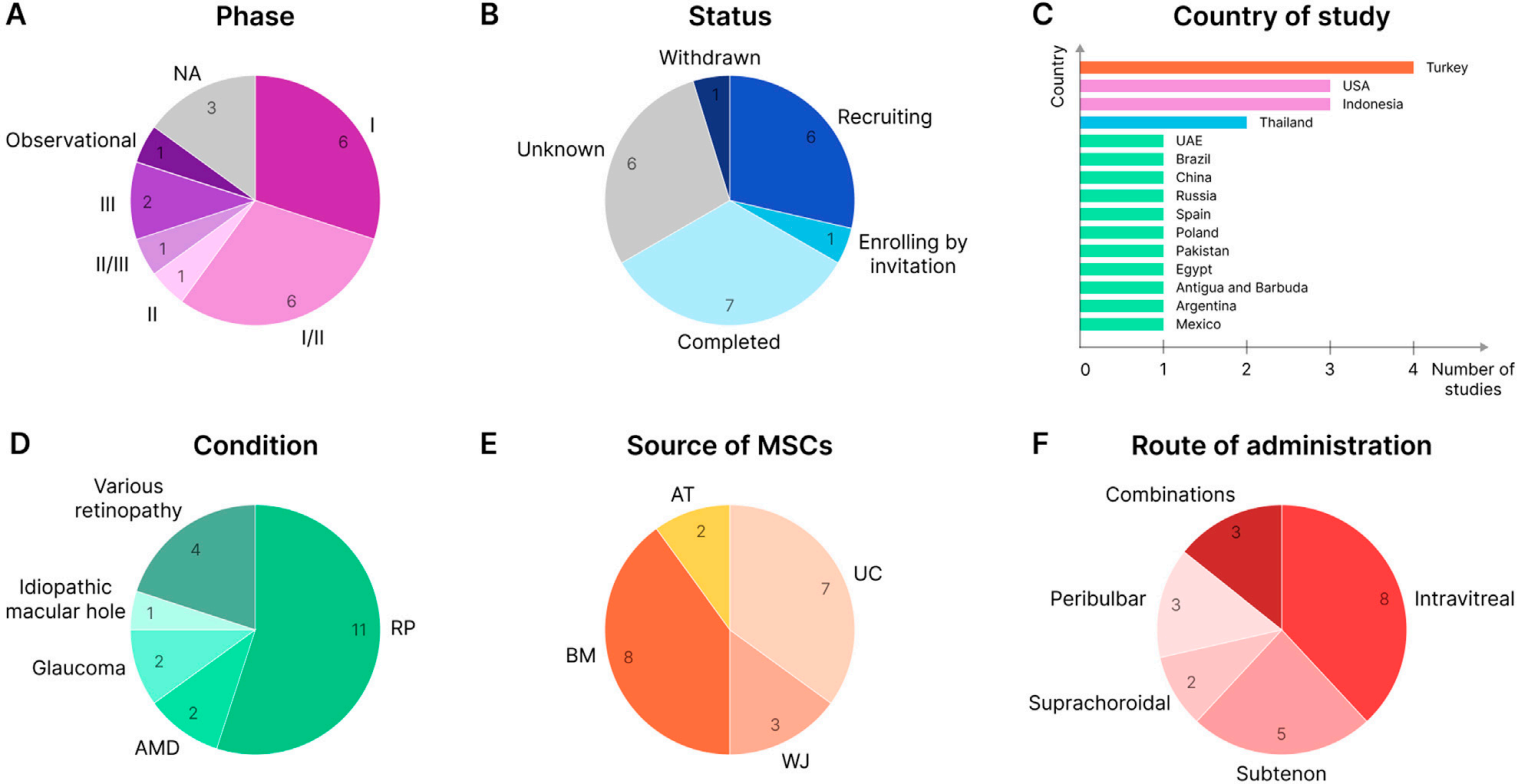
MSC-based clinical trials for retinal degenerative conditions (valid for June 2024). **(A)** Clinical trials by study phase. NA - not applicable. **(B)** Study status. **(C)** Country of study. The largest number of studies are located in Turkey, the United States, and Indonesia. **(D)** Clinical trials by the condition at which the therapy is aimed. **(E)** Clinical studies by MSC source. **(F)** Route of administration.

There are currently 20 clinical trials registered on ClinicalTrials.gov, 2024 (Table 1) in which MSCs or their derivatives are tested for therapeutic intervention in various retinal degenerative conditions. Most of these studies are in phase I or phase I/II clinical trials (Figure 5A). Several studies, such as NCT04224207 and NCT05800301, have already completed phase III trials. RP therapy with WJ-MSCs alone or in combination with retinal electromagnetic stimulation (rEMS) was found to be effective and safe (Özmert and Arslan, 2020a; Özmert and Arslan, 2020b; Özmert and Arslan, 2023). There are a total of 7 completed clinical studies (Figure 5B), which further supports the safety of MSCs for various therapeutic approaches as well as the efficacy required to complete a clinical study (Mangunsong et al., 2019; Tuekprakhon et al., 2021; Vilela et al., 2021; Özkan et al., 2023). However, one study (NCT02024269) was withdrawn due to the observed severe loss of vision (associated with retinal detachment, intraretinal hemorrhage, etc.) in patients following intravitreal injection of autologous ADSCs (Kuriyan et al., 2017).

**TABLE 1:** MSCs in clinical trials.

ClinicalTrials.gov ID of study	Phase	Status	Condition	MSCs type	Route of administration	Start and actual/expected completion dates	Country
NCT01531348	I	Completed (Tuekprakhon et al., 2021)	RP	BMSCs	Intravitreal	2012–2020	THA
NCT02016508	I/II	Unknown	AMD	BMSCs	Intravitreal	2013–2015	EGY
NCT02330978	I	Completed (Vilela et al., 2021)	Glaucoma	BMSCs	Intravitreal	2014–2016	BRA
NCT02280135	I	Completed	RP	BMSCs	Intravitreal	2014–2017	ESP
NCT01920867	NA	Unknown (Weiss et al., 2015a; Weiss et al., 2016b; Weiss et al., 2017; Weiss et al., 2015b; Weiss et al., 2016a; Weiss and Levy, 2018; Weiss and Levy, 2019a; Weiss and Levy, 2019b; Weiss and Levy, 2021)	RP, SD and other macular degenerations, glaucoma, optic atrophy, etc.	BMSCs	Retrobulbar, subtenon, intravitreal, intraocular, and intravenous (different combinations)	2012–2020	USA
NCT03011541	NA	Recruiting	RP, SD, AMD and other macular degenerations, glaucoma, optic atrophy, vision loss night, etc.	BMSCs	Retrobulbar, subtenon, intravitreal, intraocular, and intravenous (different combinations)	2016–2026	USA, UAE
NCT03772938	I	Unknown	RP, SD, BVMD, AMD	BMSCs	Intravitreal	2018–2020	POL
NCT06242379	I/II	Recruiting	RP	BMSC-derived EVs	Intravitreal	2024–2026	THA
NCT02024269	NA	Withdrawn (Kuriyan et al., 2017)	AMD	ADSCs	Intravitreal	2013–2017	USA
NCT02144103	I/II	Unknown	Glaucoma	ADSCs	Subtenon	2014–2019	RU
NCT04315025	I/II	Completed (Mangunsong et al., 2019)	RP	UC-MSCs or/and CM	Peribulbar	2018–2019	IDN
NCT05786287	Observational	Enrolling by invitation	RP	UC-MSCs or/and CM	Peribulbar	2023–2025	IDN
NCT05909488	I/II	Recruiting	RP	UC-MSCs and CM	Peribulbar	2023–2025	IDN
NCT04763369	II	Unknown	RP	UC-MSCs	Subtenon or suprachoroidal	2021–2022	PAK
NCT05147701	I	Recruiting	RP, macular degenerations, glaucoma, optic atrophy, etc.	UC-MSCs	Subtenon and intravenous	2022–2026	ATG, ARG, MEX
NCT05712148	I/II	Completed (Özkan et al., 2023)	RP	Spheroidal UC-MSCs in fibrin matrix	Suprachoroidal	2019–2022	TUR
NCT03437759	Early I	Unknown (Zhang X. et al., 2018)	Idiopathic macular hole	UC-MSC-derived EVs	Intravitreal	2017–2021	CHN
NCT04224207	III	Completed (Özmert and Arslan, 2020a; 2020b)	RP	WJ-MSCs	Subtenon	2019–2020	TUR
NCT05800301	III	Completed (Özmert and Arslan, 2023)	RP	WJ-MSCs	Subtenon + rEMS	2019–2022	TUR
NCT05413148	II/III	Recruiting	RP	WJ-MSCs and WJ-MSC-derived EVs	Subtenon	2022–2023	TUR

The largest number of MSC-based clinical studies aiming at treating retinal degenerative conditions have been reported in Turkey, the United States, and Indonesia (Figure 5C). At the same time, the largest studies in terms of patient cohorts are being conducted in the United States: the target number of participants in the Stem Cell Ophthalmology Treatment Study (SCOTS) and SCOTS2 is 300 and 500 patients, respectively (NCT01920867 and NCT03011541). Patients involved in SCOTS and SCOTS2 may have a wide range of retinal or optic nerve damage conditions: RP, SD, AMD and other macular degenerations, glaucoma, optic atrophy, etc. (Weiss et al., 2015a; Weiss et al., 2015b; Weiss et al., 2016a; Weiss et al., 2016b; Weiss et al., 2017; Weiss and Levy, 2018; Weiss and Levy, 2019a; Weiss and Levy, 2019b; Weiss and Levy, 2021). However, many other studies tend to enroll patients with a specific retinal degenerative condition such as RP, AMD, or glaucoma, with RP being the condition of interest in more than half of the known studies (Figure 5D).

BM and UC (including WJ) are most often used as a source of cells for therapy, and AT-derived MSCs are much less commonly used (Figure 5E). Autologous BMSCs and ADSCs, or allogeneic UC-MSCs and WJ-MSCs, are typically used for administration to the patient. One study (NCT05712148) uses spheroidal UC-MSCs embedded in a matrix and implanted via the suprachoroidal route (Özkan et al., 2023). Also, some studies (NCT06242379, NCT03437759, and NCT05413148) test the effects of extracellular vesicles (EVs) isolated from BMSCs, UC-MSCs, or WJ-MSCs instead of intact cells and deliver various functional therapeutic molecules (Zhang X. et al., 2018). In other studies (NCT04315025 and NCT05786287), the cell-free approach is implemented by injecting the patient with MSC-conditioned medium (CM) containing various factors (Mangunsong et al., 2019). The introduction of cells into the human eye is also carried out in various ways: intravitreal and subtenon injections are the most common; suprachoroidal, peribulbar, retrobulbar, and intravenous injections are less commonly used (and sometimes in various combinations, such as in SCOTS and SCOTS2) (Figure 5F).

Thus, MSCs are promising candidates for cell therapy of retinal degenerative diseases, including IRD, due to, among other things, their ability to differentiate into retinal cells.

## 4 Discussion

Most IRDs are caused by the dysfunction of a single gene, with different mutations in the same gene leading to distinct disease phenotypes. Currently, gene therapy treatments for these disorders are being actively developed worldwide and employ strategies for both whole gene replacement and correction of point mutations (Schneider et al., 2022; Hansen et al., 2023). The FDA approval in 2017 of Luxturna, the first and only IRD gene therapy aimed at treating LCA2 caused by mutations in the RPE65 gene, inspired researchers’ enthusiasm for developing treatments for other IRDs. Thus, there is a great need for effective modeling of IRD pathology and the testing of therapeutic agents. The biggest advantage of *in vitro* models stems from the fact that *in vivo* models for these purposes are costly and time-consuming. Moreover, there are often cases where animal models inaccurately reproduce the human disease phenotype due to anatomical and physiological differences (Slijkerman et al., 2015). In contrast, *in vitro* cell-based models save time and money, are relatively easy to use, and replicate physiological processes with varying degrees of fidelity. The application of such models in the early stages of preclinical drug development contributes to reducing animal studies in accordance with the principles of the 3Rs: replacement, reduction, and refinement of animal use for research purposes (Russell and Burch, 1959).

This review examined the various types of cells that can be used to create *in vitro* IRD models. The simplest approach involves the use of immortalized cell lines, while the most complex entails the creation of organoid cultures based on iPSCs. Each approach has its advantages and disadvantages. The better a model reproduces the *in vivo* state of cells (the higher its clinical relevance), the more laborious and costly its generation becomes, making the use of such models in large-scale experiments economically impractical. Therefore, there is currently no perfect cell source for creating *in vitro* models, and the final choice depends on the modeling goals. Yet, the mesenchymal stem cells discussed in this review more deeply are a promising cell source for developing *in vitro* models because they can differentiate into retinal cells, such as RPE cells, photoreceptor cells, and RGCs, and can be used both for modeling retinal diseases and for regenerative medicine purposes.

Significant advantages of creating *in vitro* IRD models based on MSCs include their accessibility, relative simplicity of isolation from various sources, ease of expansion and manipulation, and the ability to control their differentiation. If MSCs are isolated from a healthy donor that does not carry the IRD mutation, genetic editing and knockout of genes responsible for disease progression allow for the reproduction of pathological phenotypes. As discussed above, differentiation of MSCs into retinal cells can be achieved through various methods: culturing in induction media, culturing in conditioned media, co-culture with retinal cells, and regulation of gene expression via miRNAs or viral vectors.

Following differentiation, retinal cells are characterized by the expression of mature and immature markers. For example, commonly identified markers include RCVRN, RHO, and opsins for photoreceptor cells, and RPE65, CRALBP, and bestrophin for RPE cells. The morphology of MSCs differentiated into photoreceptor-like cells is typically described as neuron-like, with no characteristic outer and inner segments of photoreceptors usually observed. The ability of these cells to exert neuronal excitability is evaluated by their calcium response to glutamate or high potassium ion concentrations, while RPE-like cells are tested for their ability to phagocytose the outer segments of photoreceptors and secrete the neurotrophic factors BDNF and GDNF. The latter cells usually exhibit cobblestone-like morphology but often do not acquire cell pigmentation. Overall, MSC-derived retinal cells lack structural resemblance to *in vivo* cells. Moreover, these cells often demonstrate simultaneous expression of markers characteristic of various retinal cell types rather than just the target ones. Therefore, existing differentiation protocols are imperfect and require further optimization of conditions to increase the yield of target cells with a higher degree of relevance to *in vivo* cells and reduce the yield of cells with mixed marker expression. Thus, MSCs differentiated into retinal cells are characterized by an inconsistent retinal phenotype, so the quality and functionality of these cells may not fully replicate the characteristics of native retinal tissue.

The differentiation process of MSCs into retinal cells typically takes 14–28 days, depending on the chosen strategy, which is significantly less than the time required for the differentiation of 3D retinal organoids from iPSCs (up to 180 days for the expression of mature retinal neuron markers) (Afanasyeva et al., 2021). At the same time, iPSCs differentiated into retinal cells over a long period of time have a greater structural similarity to retinal cells than differentiated MSCs. This is due to the significantly restricted plasticity of MSCs compared to iPSCs, which does not allow them to fully differentiate into specific retinal cell types, such as photoreceptors, which are critical in IRD studies. Other significant disadvantages of MSCs include limited proliferation capacity, which reduces the lifespan of cells in culture; source- and donor-dependent variability, which may affect the reproducibility of studies; loss of important MSC markers in long-term cultures; and a significant decrease of their multilineage differentiation abilities. As a result, problems may arise in the use of MSCs in long-term studies and large-scale applications. Moreover, unlike iPSCs, MSCs can only be used to create 2D cell models of specific types of retinal cells due to their multipotency and inability to form self-organizing, multilayered structures (ROs). In these aspects, MSCs are undoubtedly inferior to iPSCs as a source of cells for *in vitro* IRD modeling. However, the highlighted advantages of MSCs (available sources for isolation, easier control of differentiation, etc.) more than compensate for the disadvantages. As a result, MSCs can be used to model IRD *in vitro*, being cheaper than iPSCs and more relevant than immortalized cell lines.

Individual retinal cell types can be isolated from heterogeneous populations of cells obtained during differentiation, for example, using flow cytometry. To mimic physiological conditions and, consequently, overcome some of the limitations of 2D cultures, the described technology for creating MSC-based *in vitro* models can be combined with modern “organ-on-a-chip” (microfluidics) or 3D bioprinting technologies. The “organ-on-a-chip” technology allows IRD modeling in a dynamic perfusion system, simulating complex microphysiological conditions and enabling control over the biomechanical properties of the cellular environment (Upadhyay et al., 2022; Nithin et al., 2023). Currently, this technology is successfully used in combination with iPSC-derived ROs to provide perfusion resembling blood flow in the retinal vasculature (Achberger et al., 2019). The 3D bioprinting technology can be used to create 3D structures from individual cell types, which also finds application in modeling retinal physiology and diseases (Kravchenko et al., 2023).

Clearly, the use of MSCs differentiated into retinal cells is not the only possible approach for *in vitro* IRD modeling, and, like others, this method has its advantages and disadvantages. However, while more relevant clinical models are being established, *in vitro* IRD modeling relying on MSCs is a very useful, economic, and promising platform.

## 5 Conclusion

At present, the unique properties of MSCs make them very desirable for regenerative therapy and *in vitro* modeling of degenerative retinal disorders, including IRDs. Such *in vitro* models can be used both to study IRD pathology and for drug testing. Compared to the popular pluripotent stem cells (ESCs and iPSCs), MSC isolation and subsequent differentiation into retinal cells do not come with significant ethical and practical restrictions. Moreover, the establishment of MSC-based *in vitro* models is cheaper and faster, which makes them a good alternative source for large-scale experiments, such as drug screening. Nevertheless, the technology for differentiating MSCs into retinal cells needs to be improved in the future in order to increase the yield and functionality of differentiated cells.

## Glossary

IRD: inherited retinal disease
MSCs: mesenchymal stem cells
iPSCs: induced pluripotent stem cells
BM: bone marrow
AT: adipose tissue
DT: dental tissue
UC: umbilical cord
RPE: retinal pigment epithelium
BRB: blood-retinal barrier
ONL: outer nuclear layer
INL: inner nuclear layer
GCL: ganglion cell layer
OPL: outer plexiform layer
IPL: inner plexiform layer
RGCs: retinal ganglion cells
AR: autosomal recessive
AD: autosomal dominant
XL: X-linked
MT: mitochondrial
RP: Retinitis pigmentosa
LCA: Leber congenital amaurosis
SD: Stargardt disease
BVMD: Best vitelliform macular dystrophy
NCMD: North Carolina macular dystrophy
XLRS: X-linked retinoschisis
ML: Malattia Leventinese
AMD: age-related macular degeneration
NHP: non-human primate
RDH: retinol dehydrogenase
ROs: retinal organoids
PSCs: pluripotent stem cells
ESCs: embryonic stem cells
NRL: neural retinal leucine zipper transcription factor
PDE6: phosphodiesterase 6
FGF2: basic fibroblast growth factor
EGF: epidermal growth factor
GDNF: glial cell line-derived neurotrophic factor
IGFBP5: insulin-like growth factor-binding protein 5
CTGF: connective tissue growth factor
FACS: fluorescence-activated cell sorting
MACS: magnetic-activated cell sorting
RT-DC: real-time deformability cytometry
PNA: peanut agglutinin lectin
EMT: epithelial-mesenchymal transition
CESC: retinal ciliary epithelial stem cells
RPESC: RPE stem cells
PBMCs: peripheral blood mononuclear cells
Shh: Sonic hedgehog
ISCT: International Society for Cellular Therapy
BMSCs: bone marrow-derived mesenchymal stem cell
ADSCs: adipose-derived stem cells
DPSCs: dental pulp stem cells
SHEDs: stem cells from human exfoliated deciduous teeth
PDLSCs: periodontal ligament stem cells
UC-MSCs: umbilical cord mesenchymal stem cells
WJ-MSCs: Wharton’s jelly mesenchymal stem cells
UCB-MSCs: umbilical cord blood mesenchymal stem cells
ITS: insulin-transferrin-selenium
IGF1: insulin-like growth factor 1
Dkk-1: Dickkopf-1
T3: triiodothyronine
BDNF: brain-derived neurotrophic factor
CNTF: ciliary neurotrophic factor
NGF: nerve growth factor
OM-MSCs: mesenchymal stem cells derived from olfactory mucosa
CJMSCs: conjunctiva mesenchymal stem cells
PLLA: poly-l-lactic acid
PCL: polycaprolactone
PEG: polyethylene glycol
VIP: vasoactive intestinal peptide
POS: photoreceptor outer segment
miRNA: microRNA
mRNA: messenger RNA
IM: induction medium
MSH: melanocyte-stimulating hormone
TF: transcription factor
ZFN: zinc finger nuclease
TALEN: transcription activator-like effector nuclease
gRNA: guide RNA
NHEJ: non-homologous end joining
HDR: homology-directed repair
GFP: green fluorescent protein
EPO: erythropoietin
rEMS: retinal electromagnetic stimulation
SCOTS: Stem Cell Ophthalmology Treatment Study
EVs: extracellular vesicles
CM: conditioned medium
