# Supplementary material for: Evaluation of mesenchymal stem cells as an in vitro model for inherited retinal diseases
Source: Front Cell Dev Biol. 2024 Nov 15;12:1455140. doi: 10.3389/fcell.2024.1455140 (PMC11604642; doi:10.3389/fcell.2024.1455140)
Supplement: Supplementary file 1 [file Table1.DOCX]

Supplementary Material

Supplementary Table 1. Methods of MSCs differentiation into retinal cells

| **Source of MSC isolation** | **Phenotyping markers** | **Differentiation markers** | **Differentiation method** | **Result of differentiation** |
| --- | --- | --- | --- | --- |
| Bone marrow of adult human (Chiou et al., 2005) | CD34-, CD44-, CD45-, cKit-, flk-1-, MHCI-, MHCII-, CD13+, CD49b+ | NES, opsin, PKC | **Stage 1.** Cells at passage 8 were cultured in IM1 (DMEM/F12, 0.6% glucose, 25 μg/ml insulin, 100 μg/ml transferrin, 20 nM progesterone, 60 μM putrescine, 30 nM selenium chloride, 2 mM glutamine, 3 mM sodium bicarbonate, 5 mM Hepes, 2 μg/ml heparin, 20 ng/ml EGF, and 20 ng/ml FGF2) for 14 days.  **Stage 2.** Neurospheres were co-cultured with UV-inactivated human RPE cells on a gelatin-coated dish for 21 days. | Photoreceptor-like cells (morphology of neuronal cells, K+-evoked calcium responses) |
| Bone marrow of adult human (Duan et al., 2013) | CD14-, CD34-, CD45-, CD73+, CD90+, CD105+ | PAX6, MITF, OTX2, bestrophin, tyrosinase, PMEL17, RPE65, ZO-1, PEDF, CRALBP | Cells were co-cultured with pig RPE cells in a transwell system in DMEM/F12 medium supplemented with 10% FBS for 14 days. | RPE-like cells (intracellular pigment granules, apical microvilli, POS-phagocytosis, secretion of BDNF and GDNF) |
| Bone marrow of rat (Kadkhodaeian et al., 2019b) | CD34-, NES-, GFAP-, CD44+, CD90+, CD166+, fibronectin+ | OCT4, SOX2, NANOG, NES, OTX2, RPE65, CRALBP | **Stage 1.** Cells at passage 3 were cultured in IM1 (DMEM/F12, 2 % B27, 20 ng/ml of EGF, 20 ng/ml FGF2) for 2 days.  **Stage 2.** Neurospheres were cultured in IM2 (DMEM low glucose, 1% FBS, 14 nM selenious acid, 28 nM hydrocortisone, 300 nM linoleic acid, 830 nM insulin, 63 nM transferrin, 2.4 μM putrescine, 0.01 nM T3) for 15 days. | RPE-like cells (pigmented spheres capable of cell monolayer formation and POS-phagocytosis) |
| Bone marrow of rabbit (Choi et al., 2019) | - | RPE65, NPR-A, CRALBP | Cells were co-cultured with UV-inactivated ARPE-19 cells on a gellan-gum hydrogel in medium supplemented with 1х B27 for 21 days. | RPE-like cells (epithelial polygonal shape, increased proliferative ability) |
| Adipose tissue of adult human (Vossmerbaeumer et al., 2009) | CD14-, CD34-, CD45-, HLA‐2-, CD133-, CD144-, CD29+, CD44+, CD73+, CD90+, CD105+, HLA‐1+, CD106+ | PAX6, MAP2, PEDF, bestrophin, tyrosinase, RPE65, CK8/18 | Cells were cultured for 5-8 days in different medium:  - conditioned for 7 days with pig RPE cells or human RPE cells;  - containing 5 μM vasoactive intestinal peptide (VIP);  - conditioned for 7 days with pig RPE cells and containing 5 μM VIP. | RPE-like cells (MSH-induced formation of pigmented granules) |
| Adipose tissue of adult human (Rezanejad et al., 2014) | CD34-, CD45-, CD73+, CD90+, CD105+ | NES, PAX6, CHX10, RPE65, CRALBP, CK8/18, CRX, NRL, RCVRN, phosducin, RHO | Cells were transduced with lentiviral vector carrying the human PAX6(5a) gene and cultured in DMEM/F12 medium supplemented with 20% FBS and fibronectin for up to 6 days. | RPE- and photoreceptor-like cells (morphology of neuronal cells, connecting network between cells) |
| Adipose tissue of adult human (Zhang et al., 2017) | CD40-, CD133-, CD73+, CD90+, CD105+. | Bestrophin, RPE65, CK8 | Cells were cultured for 10 days in DMEM/F12 medium conditioned with ARPE19. | RPE-like cells (increased proliferative and migratory ability) |
| Adipose tissue of adult human (Huang Y. et al., 2018) | CD14-, CD34-, CD45-, CD44+, CD73+, CD90+, CD105+ | PAX6, NES, CRX, NRL, RHO, RCVRN, ATOH7, TUBB3, POU4F2 | **Stage 1.** Cells at passage 3-5 were cultured in IM1 (DMEM/F12, 10% knockout serum replacement, 1x B27, 1 ng/ml Noggin, 1 ng/ml Dkk-1, 5 ng/ml IGF1) in ultralow attachment culture dish for 3 days.  **Stage 2.** Neurospheres were cultured in IM2 (DMEM/F12, 1x B27, 1x N2, 10 ng/ml Noggin, 10 ng/ml Dkk-1, 10 ng/ml IGF1, 5 ng/ml FGF2) in Matrigel-coated culture dish for 7 days, then in IM3 (DMEM/F12, 1x B27, 1x ITS, 10 ng/ml Noggin, 10 ng/ml Dkk-1, 10 ng/ml IGF1, 5 ng/ml FGF2 + 20 μM JAG1/10 μM DAPT) for 14 days. | Photoreceptor- and RGC-like cells (morphology of neuronal cells, glutamate-evoked calcium responses) |
| Adipose tissue of adult human (Kadkhodaeian et al., 2019a) | CD34-, CD11b+, CD90+, CD105+ | RPE65, CRALBP | Cells at passage 4 were first cultured in DMEM/F12 medium for 2 days, then in  IM (DMEM, 1% FBS, 2 mM L-glutamine, 0.01 nM T3, 2 μg/ml heparin, 830 nM insulin, 20 ng/ml EGF) on a gelatin-coated coverslip for 80 days. | RPE-like cells (epithelial polygonal shape, intracellular pigment granules, apical microvilli, tight intercellular junctions) |
| Adipose tissue of adult human (Ling et al., 2023) | CD14-, CD34-, CD45-, CD44+, CD73+, CD90+, CD105+ | PAX6, CHX10, LHX2, SOX2, RX, NES, CRX, NRL, RCVRN, RHO, ATOH7, TUBB3 | **Stage 1.** Cells at passage 3-5 were cultured in IM1 (DMEM/F12, 10% knockout serum replacement, 1x B27, 1 ng/ml Noggin, 1 ng/ml Dkk-1, 5 ng/ml IGF1) in ultra-low attachment culture dish for 3 days.  **Stage 2.** Neurospheres were cultured in IM2 (DMEM/F12, 1x B27, 1x N2, 100 ng/ml Noggin, 10 ng/ml Dkk-1, 100 ng/ml IGF1, 50 ng/ml FGF2) in ultra-low attachment culture dish or Matrigel-coated culture dish for 21 days.  Also, in one of the differentiation options, cells were cultured in DMEM/F12 medium supplemented with 5% FBS in ultra-low attachment culture dish for 24 days. | Photoreceptor- and RGC-like cells (morphology of neuronal cells) |
| Periodontal ligament of adult human (Huang L. et al., 2013) | Notch1, BMP2, Slug, Snail, NES, Tuj1, p75/NGFR, CD44, CD90, vimentin, c-Myc, Klf4, NANOG, SSEA4 | DCX, LHX2, CHX10, RX, SOX2, OTX2, NRL, RHO, GRK1 | **Stage 1.** Cells at passage 3 were cultured in IM1 (DMEM/F12, 1% B27, 1 ng/ml Noggin, 1 ng/ml Dkk-1) in ultra-low attachment culture plate for 3 days.  **Stage 2.** Neurospheres were cultured in IM2 (IM1, 1x N2, 10 ng/ml Noggin, 10 ng/ml Dkk-1) in Matrigel-coated culture plate for up to 45 days. | Photoreceptor-like cells (morphology of neuronal cells, connecting network between cells, glutamate-evoked calcium responses) |
| Periodontal ligament of adult human (Ng et al., 2015) | Notch1, BMP2, Slug, Snail, NES, Tuj1, p75/NGFR, CD44, CD90, vimentin, c-Myc, Klf4, NANOG, SSEA4 | PAX6, CHX10, ATOH7, POU4F2, TUBB3, MAP2, TAU, NEUROD1, SIX3 | **Stage 1.** Cells at passage 3-5 were cultured in IM1 (DMEM/F12, 10% knockout serum replacement, 1x B27, 1 ng/ml Noggin, 1 ng/ml Dkk-1, 5 ng/ml IGF1) at low adherent culture for 3 days.  **Stage 2.** Neurospheres were cultured in IM2 (DMEM/F12, 1x B27, 1x N2, 100 ng/ml Noggin, 10 ng/ml Dkk-1, 100 ng/ml IGF1, 50 ng/ml FGF2) in laminin-coated dishes for 7 days, then in IM3 (DMEM/F12, 1x B27, 1x ITS, 10 ng/ml Noggin, 10 ng/ml Dkk-1, 10 ng/ml IGF1, 50 ng/ml FGF2, 10 ng/ml BDNF, 10 ng/ml CNTF, 10 ng/ml NGF, 10 ng/ml Shh) for 14 days. | RGC-like cells (morphology of neuronal cells, glutamate-evoked calcium responses, spontaneous electrical activities) |
| Dental pulp of rat (Roozafzoon et al., 2015) | CD31-, CD34-, CD45-, CD44+, CD73+, CD90+, CD105+ | PAX6, ATOH7, MAP2, POU4F2, GFAP | **Stage 1.** Cells were cultured in IM1 (DMEM/F12, 0.5% FBS, 1% N2, 2 μg/ml heparin, 10 ng/ml FGF2) in a dish coated with 150 μg/ml poly-D-Lysine and 1 μg/ml Laminin or in fibrin gel for 11 days.  **Stage 2.** Medium was altered to the IM2 (DMEM/F12, 0.5% FBS, 500 ng/ml Shh, 8 ng/ml FGF2) and cultivation continued for 16 hours. | RGC-like cells (morphology of neuronal cells) |
| Exfoliated deciduous teeth of human (Li X. et al., 2019) | CD34-, CD45-, CD73+, CD90+, CD105+, CD146+, TUBB3+, NES+, GFAP+ | NEUROD1, ASCL1, TAU, GluR2, NES, OTX2, AIPL1, PAX6, RX, CHX10, CRX, NRL, RCVRN, RHO, OPN1SW | **Stage 1.** Cells at passage 3-5 were cultured in IM1 (DMEM/F12, 10% knockout serum replacement, 1x B27, 1 ng/ml Noggin, 1 ng/ml Dkk-1, 5 ng/ml IGF1) in a low-attachment dish for 3 days.  **Stage 2.** Neurospheres were dissociated and cultured in IM2 (DMEM/F12, 1x B27, 1x N2, 100 ng/ml Noggin, 10 ng/ml Dkk-1, 50 ng/ml IGF1, 20 ng/ml FGF2) in Matrigel-coated culture dish for 7 days, then in IM3 (DMEM/F12, 1x N2, 1x ITS, 10 ng/ml Noggin, 10 ng/ml Dkk-1, 10 ng/ml IGF1, 20 ng/ml FGF2, 20 ng/ml Shh, 40 ng/ml T3, 500 nM all-trans retinoic acid) for 14 days. | Photoreceptor-like cells (morphology of neuronal cells, connecting network between cells, K+/glutamate-evoked calcium responses) |
| Olfactory mucosa of adult human (Lu et al., 2017) | CD34-, CD45-, CD73+, CD90+, CD105+ | RHO | Cells at passage 4 were seeded in DMEM/F12 medium supplemented with 10% FBS in coverslips coated with poly-D-lysine, when cells grew to 80% confluence, 100 ng/ml EGF, 50 μM taurine, and 0.5 μM all-trans retinoic acid were added to the medium and cultured for 10 days. | Photoreceptor-like cells (morphology of neuronal cells, connecting network between cells) |
| Conjunctival tissue of adult human (Nadri et al., 2013) | CD11b-, CD34-, CD38-, CD45-, CD83-, CD86-, CD133-, flk1-, CD13+, CD29+, CD44+, CD73+, CD105+, CD166+ | NES, MAP2, CRX, PKC, RHO, GFAP | Cells were seeded onto poly-l-lactic acid (PLLA) random/ aligned nanofibrous scaffolds and cultured in IM (DMEM, 2% FBS, 50 μM taurine) for 14 days. | Photoreceptor-like cells |
| Conjunctival tissue of adult human (Nadri et al., 2017) | CD11b-, CD34-, CD38-, CD45-, CD83-, CD86-, CD133-, flk1-, CD13+, CD29+, CD44+, CD73+, CD105+, CD166+ | RCVRN, RHO | Cells were seeded onto poly caprolactone and poly ethylene glycol (PEG/PCL) scaffolds included or no 50 μM taurine and cultured in DMEM medium supplemented with 2% FBS or in IM (DMEM, 2% FBS, 50 μM taurine) for 21 days. | Photoreceptor-like cells |
| Conjunctival tissue of adult human (Ranjbarnejad et al., 2019) | - | RCVRN, RHO | Cells were transduced with lentiviruses containing let-7a miRNA and cultured for up to 42 days. | Photoreceptor-like cells |
| Conjunctival tissue of adult human  (Rahmani et al., 2020) | CD11b-, CD34-, CD38-, CD45-, CD83-, CD86-, CD133-, flk1-, CD13+, CD29+, CD44+, CD73+, CD105+, CD166+ | PKC, RCVRN, RHO | Cells were transduced with lentiviruses containing miRNA-9 and seeded onto silk fibroin-poly-L-lactic acid (SF-PLLA) nanofibrous scaffold for up to 21 days. | Photoreceptor-like cells |
| Conjunctival tissue of adult human (Naderi and Nadri, 2022) | CD11b-, CD34-, CD38-, CD45-, CD83-, CD86-, CD133-, flk1-, CD13+, CD29+, CD44+, CD73+, CD105+, CD166+ | PKC, PRPH, RCVRN, RHO | Cells were transduced with lentiviruses containing miRNA-9, cultivated on the scaffold obtained by polymerization of silk fibroin and reduced graphene oxide nanoparticles (SF-rGo), then electrical induction was implied and cells were cultured for 7 days. | Photoreceptor-like cells |
| Amniotic tissue of human (Choi et al., 2015) | - | MITF, OTX2, RPE65, bestrophin, EMMPRIN | Cells were transfected four times at days 0, 3, 10 and 17 with anti-miR-410 at a final concentration of 30 nM and cultured for up to 21 days. | RPE-like cells (epithelial polygonal shape, phagocytic ability) |
| Amniotic tissue of human (Choi et al., 2016) | CD14-, CD31-, CD34-, CD45-, CD4+, CD24+, CD29+, CD49b+, CD73+, CD90+, CD105+ | RX, CRX, THRB, NR2E3, NRL, PRKCA, STX1A, CALB2, POU4F2, OPN1MW | Cells at passage 2-5 were cultured in the differentiation medium containing N2, B27 and 1% FBS, transfected every 7 days up to three times with anti-miR-203 at a final concentration of 30 nM and cultured for up to 28 days. | Photoreceptor-like cells (morphology of neuronal cells) |
| Umbilical cord blood of human (Choi et al., 2016) | CD10-, CD14-, CD31-, CD33-, CD34-, CD45-, CD62p-, CD133-, CD24+, CD29+, CD44+, CD73+, CD90+, CD105+ | CHX10, OTX2, THRB, NR2E3, NRL, PRKCA, RHO, CALB2, POU4F2, OPN1MW | Cells at passage 8-15 were cultured in the differentiation medium containing N2, B27 and 1% FBS, transfected every 7 days up to three times with anti-miR-203 at a final concentration of 30 nM and cultured for up to 28 days. | Photoreceptor-like cells (morphology of neuronal cells) |
| Umbilical cord blood of human (Choi et al., 2017) | CD10-, CD14-, CD31-, CD33-, CD34-, CD45-, CD62p-, CD133-, CD24+, CD29+, CD44+, CD73+, CD90+, CD105+ | MITF, LRAT, RPE65, bestrophin, EMMPRIN | Cells were transfected four times at days 0, 3, 10 and 17 with anti-miR-410 at a final concentration of 30 nM and cultured for up to 21 days. | RPE-like cells (phagocytic ability) |
| Umbilical cord of human (Zhu X. et al., 2022) | CD34-, CD45-, MHCII-, CD29+, CD44+, CD73+, CD90+, CD105+ | RPE65, MERTK, TYRP1, CRALBP, PEDF, ZO-1 | Cells were transduced with an equal ratio of a combination of retroviruses carrying 5 TF (CRX, NR2E1, C-MYC, LHX2, SIX6) and cultured in DMEM/F12 medium supplemented with 10% FBS for 7 days. | RPE-like cells (epithelial polygonal shape, apical microvilli,POS-phagocytosis, epithelial polarity, low cellular permeability, resistance to EMT induced by TGF-β) |
| Wharton's jelly of human (Ding et al., 2019) | CD14-, CD34-, CD45-, CD80-, CD86-, CD29+, CD44+, CD73+, CD90+, CD105+, HLA-ABC+ | CRX, RHO | Cells were transduced with lentivirus carrying EPO and cultured in IM (DMEM/F12, 3% FBS, 50 μM taurine) for up to 21 days. | Photoreceptor-like cells (morphology of neuronal cells, connecting network between cells) |
| Wharton's jelly of human (Chang et al., 2022) | CD34-, CD45-, CD29+, CD44+, CD73+, CD105+, HLA-ABC+ | MITF, OTX2, RPE65, bestrophin, PEDF, PME17, CRALBP, ZO-1 | Cells were co-cultured with ARPE19 in a transwell system in DMEM/F12 medium for 14 days. | RPE-like cells (phagocytic ability, secretion of BDNF and GDNF) |
